# Resolving species boundaries in a recent radiation with the Angiosperms353 probe set: the *Lomatium packardiae/L. anomalum* clade of the *L. triternatum* (Apiaceae) complex

**DOI:** 10.1002/ajb2.1676

**Published:** 2021-06-08

**Authors:** Michael V. Ottenlips, Donald H. Mansfield, Sven Buerki, Mary Ann E. Feist, Stephen R. Downie, Steven Dodsworth, Félix Forest, Gregory M. Plunkett, James F. Smith

**Affiliations:** ^1^ Department of Biological Sciences Boise State University Boise ID 83725 USA; ^2^ College of Idaho Caldwell ID 83605 USA; ^3^ University of Wisconsin‐Madison Madison WI 53706 USA; ^4^ Department of Plant Biology University of Illinois at Urbana‐Champaign Urbana IL 61801 USA; ^5^ Royal Botanic Gardens, Kew Richmond Surrey TW9 3AE UK; ^6^ School of Life Sciences University of Bedfordshire Luton LU1 3JU UK; ^7^ Cullman Program for Molecular Systematics New York Botanical Garden 2900 Southern Boulevard Bronx NY 10458 USA

**Keywords:** Angiosperms353, coalescence, incipient speciation, incomplete lineage sorting, incongruence, STACEY

## Abstract

**Premise:**

Speciation not associated with morphological shifts is challenging to detect unless molecular data are employed. Using Sanger‐sequencing approaches, the *Lomatium packardiae/L. anomalum* subcomplex within the larger *Lomatium triternatum* complex could not be resolved. Therefore, we attempt to resolve these boundaries here.

**Methods:**

The Angiosperms353 probe set was employed to resolve the ambiguity within *Lomatium triternatum* species complex using 48 accessions assigned to *L. packardiae*, *L. anomalum*, or *L. triternatum*. In addition to exon data, 54 nuclear introns were extracted and were complete for all samples. Three approaches were used to estimate evolutionary relationships and define species boundaries: STACEY, a Bayesian coalescent‐based species tree analysis that takes incomplete lineage sorting into account; ASTRAL‐III, another coalescent‐based species tree analysis; and a concatenated approach using MrBayes. Climatic factors, morphological characters, and soil variables were measured and analyzed to provide additional support for recovered groups.

**Results:**

The STACEY analysis recovered three major clades and seven subclades, all of which are geographically structured, and some correspond to previously named taxa. No other analysis had full agreement between recovered clades and other parameters. Climatic niche and leaflet width and length provide some predictive ability for the major clades.

**Conclusions:**

The results suggest that these groups are in the process of incipient speciation and incomplete lineage sorting has been a major barrier to resolving boundaries within this lineage previously. These results are hypothesized through sequencing of multiple loci and analyzing data using coalescent‐based processes.

Speciation is frequently associated with morphological change, especially in reproductive characters such as flowers and fruits in angiosperms (Kay et al., 2001; Givnish, 2010). However, morphological changes do not necessarily occur in reproductive characters, and in cryptic speciation, speciation events may not be associated with any discernible morphological change (Paris et al., 1989). Similarly, when the speciation process is recent or ongoing (incipient speciation), substantial morphological differences may not have had time to accrue, leading to lineages that can be difficult to distinguish morphologically (Qiu et al., 2009; Smith et al., 2018b).

DNA‐based characters are commonly used to increase the data available for species delimitation and phylogenetic reconstruction (Duminil and Di Michele, 2009; Hu et al., 2015; Herrera and Shank, 2016); their application is especially vital in cases of cryptic, recent, and incipient speciation (*Deinandra*: Baldwin, 2007; *Metrosideros*: Stacy et al., 2014; *Columnea*: Smith et al., 2018b). Over the last decade, high‐throughput sequencing (HTS) approaches have resulted in a proliferation of types and amounts of molecular data available to researchers beyond traditional Sanger sequencing (Metzker, 2010; Hipp et al., 2014; Eaton et al., 2015; Crowl et al., 2017; Bogarín et al., 2018; Stoughton et al., 2018; Stubbs et al., 2018b, 2018a,2018b, 2018a; Balao et al., 2020; Larridon et al., 2020). While these new approaches using high‐throughput molecular data have been effective at resolving recalcitrant evolutionary relationships at both deep and shallow taxonomic ranks, these data sources are not without their own challenges. Incongruencies between molecular data and morphology, ecology, and geography can result from many factors, including paralogous loci, incomplete lineage sorting (ILS), and ongoing gene flow among populations (Jacobs et al., 2018; Schuster et al., 2018; Alda et al., 2019; Kleinkopf et al., 2019; Siu‐Ting et al., 2019; Weber et al., 2019; Larridon et al., 2020; Smith and Hahn, 2020; Thomas et al. 2021b).

Target‐enrichment protocols that are not taxon specific use exons or other highly conserved regions that are relatively easy to align, ensuring homology across disparate taxa (Faircloth et al., 2015; Léveillé‐Bourret et al., 2017; Johnson et al., 2018). However, these highly conserved regions may not contain sufficient variation to be suitable for studies at the population level. Recent bioinformatics developments (Johnson et al., 2016) allow for increased ease of extracting flanking intronic regions, which are theoretically more variable and useful at shallow taxonomic levels. However, to our knowledge, the usefulness of targeted‐enrichment approaches based on universal probe sets for population‐level studies remains to be fully demonstrated practically.

Speciation is an intricate and ongoing process influenced by a variety of biotic and abiotic factors. The delimitation of species can be complex and thus should use data from a variety of sources (molecular, morphological, ecological, and geographic), considering not only evolutionary processes such as introgression and lineage sorting, but also the nature of the species unit itself. Species concepts range from similarity‐based definitions, such as the morphological species concept (Burger, 1975), to ancestry‐based concepts that incorporate the complex nature of gene inheritance, such as the genealogical species concept, which defines species as monophyletic groups that “reside at the reticulate/divergent boundary” (Baum and Shaw, 1995, p. 291). The monophyly of most species has not been tested using molecular data, and many morphological characters used to delimit species may not be taxonomically informative from an ancestry‐based perspective.

The perennial endemic North America (PENA) clade of Apioideae (Apiaceae) is one of the largest land plant radiations in western North America with 19 genera and ca. 230 species (Sun et al., 2004; Feist et al., 2017). The group has been shown to comprise several examples of apparent homoplasy in characters that were previously thought to be taxonomically informative (Downie et al., 2002; Sun and Downie, 2010a; George et al., 2014). Mature fruit characters, such as dorsal wings and number of vittae or oil tubes, have traditionally been used to delimit taxa at both the specific and generic level in this group (Coulter and Rose, 1900). *Lomatium,* the group’s largest genus (~100 spp.), has traditionally been distinguished from the closely related *Cymopterus* based on its lack of a dorsal rib wing on mature mericarps (Hitchcock and Cronquist, 1973). Sequence data from the ITS region suggested that *Cymopterus,* as traditionally described, is polyphyletic (Sun et al., 2004; Sun and Downie, 2010a,b). Moreover, the discovery that a species traditionally placed in *Lomatium* due to the lack of dorsal fruit wings falls within a clade otherwise restricted to *Cymopterus glomeratus* (Nutt.) DC. demonstrates that fruit morphology can be homoplastic (George et al., 2014). The discovery that the two species of *Orogenia* are nested within *Lomatium* was also based on results from molecular data (Sun et al., 2010a,b; George et al., 2014; Feist et al., 2017). *Orogenia* had been defined on the basis of fruits with lateral wings that were thick and corky (Coulter and Rose, 1900); however, the two species were not monophyletic within *Lomatium* based on DNA sequence data and provide another example of morphologically based taxonomy that did not reflect evolutionary history in this clade (Sun and Downie, 2010a; George et al., 2014; Feist et al., 2017). Homoplasy in fruit characters is known throughout Apiaceae, although Liu et al. (2006) found that developmental origin of the wings, carpel shape, presence of vittae, woodiness of the endocarp, position of crystals, and type of carpophores in fruits of 18 genera of Apiales were taxonomically informative. Since molecular studies have revealed such a high degree of homoplasy in traditionally taxonomically informative characters, revisiting existing species boundaries with the addition of molecular data in a phylogenomic approach is warranted throughout the PENA clade, and morphology, ecology, and geography can be mapped onto recovered clades to help determine their taxonomic ranks.

Recent investigations into the *Lomatium triternatum* (Pursh) J.M.Coult. & Rose species complex have revealed a suite of homoplasies and incongruencies between morphological and molecular data (Smith et al., 2018a). *Lomatium simplex* (Nutt. ex S.Watson) J.F.Macbr., originally described as *Peucedanum simplex* Nutt. ex S.Watson, and more recently considered a variety of *L. triternatum*, has been elevated to the rank of species within *Lomatium* because it constitutes a distinct evolutionary lineage from the rest of the *L. triternatum* complex (Smith et al., 2018a). The triternate leaf morphology most likely represents another case of convergence within the PENA clade. *Lomatium triternatum* as described in *Intermountain Flora: Vascular Plants of the Intermountain West* (Cronquist et al., 1997) is distributed throughout the northwestern United States, neighboring Canadian provinces, and the northern Great Basin and Rocky Mountains. The *Lomatium anomalum/L. packardiae* subcomplex is part of the larger *L. triternatum* complex and consists of two formerly accepted taxa with different morphologies, distributions, and ecological preferences: *Lomatium packardiae* Cronquist was originally described from material growing exclusively on “volcanic ash that has not disintegrated into clay” and with a morphology similar to *L. triternatum* var. *triternatum* except for leaflets with a different aspect (Cronquist, 1992, p. 75). *Lomatium anomalum* M.E. Jones ex J.M.Coult. & Rose has been treated at several taxonomic levels, including as a distinct species and as either a variety or subspecies of *L. triternatum*. The leaflets of *L. anomalum* are substantially wider than those of any other described entity in the complex, making it readily identifiable in the field (Hitchcock and Cronquist, 1973; Cronquist et al., 1997). It is frequently found in more mesic habitat types associated less with sagebrush and more with prairie‐like conditions. While many populations of *L. anomalum* are readily differentiated based on morphology, there are populations and individuals that exhibit both wider *L. anomalum‐*like leaves and narrower *L. triternatum* var. *triternatum* or *L. packardiae‐*type leaves. For example, individuals from the Camas Prairie populations tend to have leaflets that average over 8 mm wide, but *Ottenlips 65* and *Ottenlips 69* are 5 and 4 mm wide on average, respectively (Appendix S1), putting them in the range of the narrower leaflets found in Hell’s Canyon (*Ottenlips 57*, 5 mm wide on average) and Mann Creek (*Stevens 123*, 4.8 mm wide on average; Appendix S1). Even when taking into account morphology, ecology, and geography, the *L. packardiae/L. anomalum* subcomplex is taxonomically confusing.

To untangle this confusion, Smith et al. (2018a) used a set of seven loci (two nuclear ribosomal and five plastid) to investigate species boundaries in the larger *L. triternatum* species complex. While this approach helped to resolve some taxonomic problems, such as *L. simplex* and *L. andrusianum* (Stevens et al., 2018), this study did not recover monophyletic groups that corresponded to the morphological, ecological, and geographic patterns exhibited by the *L. anomalum/L. packardiae* subcomplex. For example, plants with wide leaflets (Fig. 1A) from prairies in central‐western Idaho formed a clade with plants that have narrow leaflets (Fig. 1E) found in and around ash beds in southeastern Oregon (Smith et al., 2018a).

**FIGURE 1:**
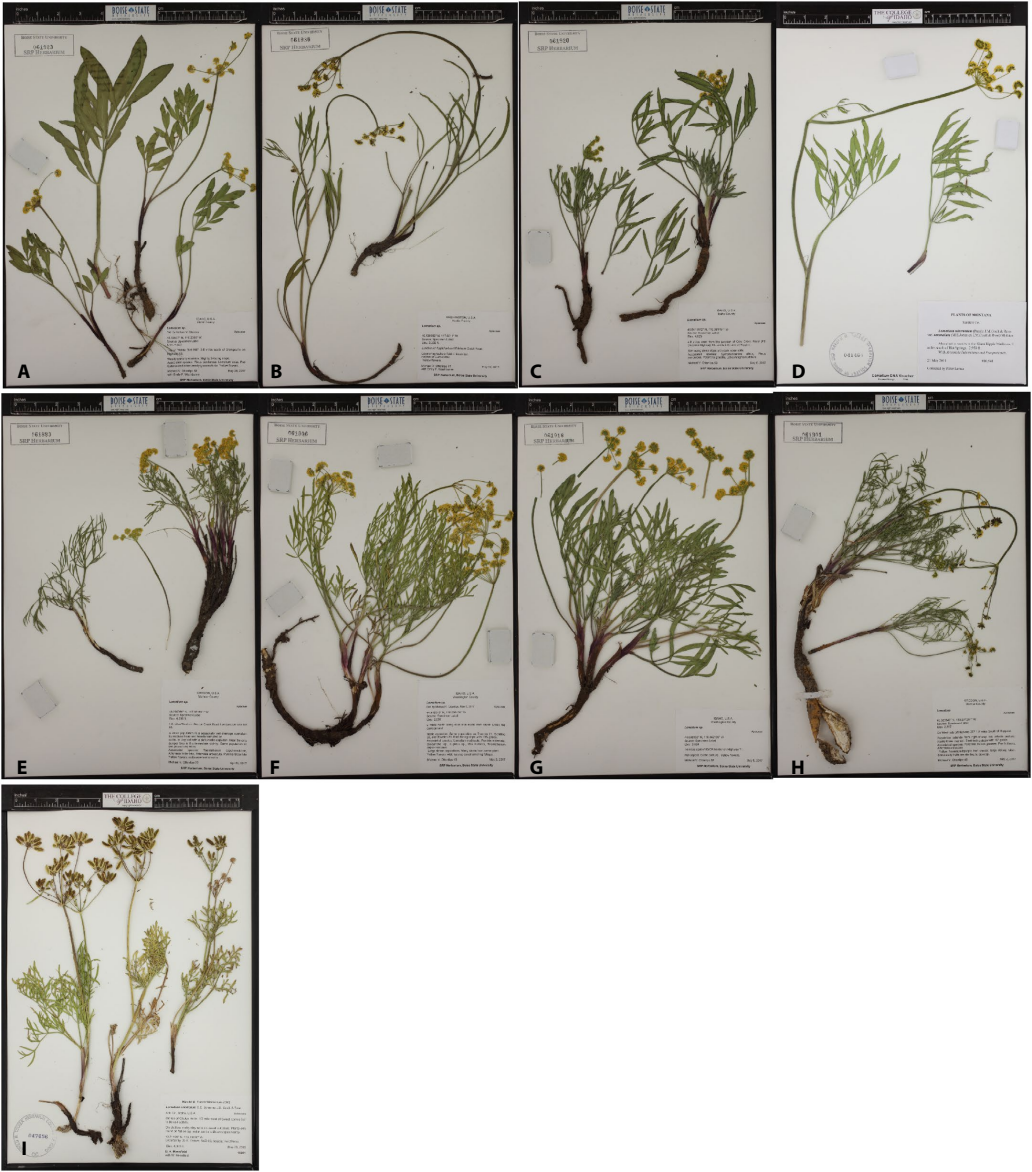
Representative herbarium specimens of the clades uncovered in the STACEY analysis (see Results). The northern clade on the top row and the southern clade on the middle row. *Lomatium andrusianum* is alone on the bottom row. Further details on vouchers are presented in Appendix S2. (A) Camas Prairie (*Ottenlips 62*). (B) *Lomatium triternatum* var. *triternatum* (*Ottenlips 77*). (C) *Ottenlips 59*. (D) Western Montana (*Lesica 10541*). (E) *Lomatium packardiae* (*Ottenlips 22*). (F) Mann Creek (*Ottenlips 45*). (G) Hell’s Canyon (*Ottenlips 57*). (H) East‐Central Oregon (*Ottenlips 40*). (I) *Lomatium andrusianum* (*Mansfield 16031*).

The observed incongruencies between results based on molecular data and expectations based on geography, ecology, and morphology could be attributed to two alternative hypotheses: (1) individuals within the *L. anomalum/L. packardiae* subcomplex are highly variable morphologically and/or phenotypically plastic, and different morphologies reflect varying environmental conditions, or (2) the taxa may be distinct and monophyletic, and the differences between the observed and expected trees from the Sanger‐based data set may be explained by historic evolutionary processes such as ILS and introgression. Introgression seems a less‐plausible explanation because there is no evidence of hybridization in this group. Smith et al. (2018a) used both plastid and nuclear ribosomal loci and did not detect any hard incongruence between datasets. Although full analyses to test hybridization using morphologies have not been explored, examination of specimens of sympatric species do not indicate any morphological intermediacy (D. H. Mansfield, J. F. Smith, personal observations). The goal of this study was to use molecular, ecological, geographic, and morphological data to determine species boundaries and understand the role of complex evolutionary scenarios and phenotypic variation in the evolution of the *L. anomalum/packardiae* subcomplex. We aimed to resolve monophyletic groups using molecular data and to investigate whether geographic, morphological, or ecological data can be diagnostic for these monophyletic groups. The genealogical species concept (Baum and Shaw, 1995) was employed, using the molecular‐based phylogenetic tree to identify monophyletic groups.

## MATERIALS AND METHODS

### Sample collection

Populations in the *L. packardiae/L. anomalum* species complex were visited between April and July during the 2017 and 2018 growing seasons to obtain fresh leaf material for molecular methods, to collect ecological data, and to acquire herbarium specimens for morphometric analysis. Prior to collecting, known localities of populations within the species subcomplex were identified using data available from the Consortium of Pacific Northwest Herbaria (http://www.pnwherbaria.org/). Additional populations were sampled opportunistically en route to known locations. Based on preliminary results available from Sanger sequencing and morphological data (see Smith et al., 2018a), several areas of known genetic diversity and/or sources of taxonomic confusion (notably the Succor Creek drainage of eastern Oregon; the Camas Prairie outside Grangeville, Idaho, USA; and Asotin County in southeastern Washington, USA) were sampled more heavily to obtain sufficient material to address taxonomic questions. Individuals of uncertain identity were identified using *Flora of the Pacific Northwest* (Hitchcock and Cronquist, 2018) and/or *Intermountain Flora* (Cronquist et al., 1997). At each location, fresh leaf material was dried in silica gel. Herbarium vouchers of representative plants were collected and deposited at SRP with duplicates at CIC (herbarium codes following Thiers, 2021; full sampling records for each analysis can be found in Appendix S2).

**FIGURE 2:**
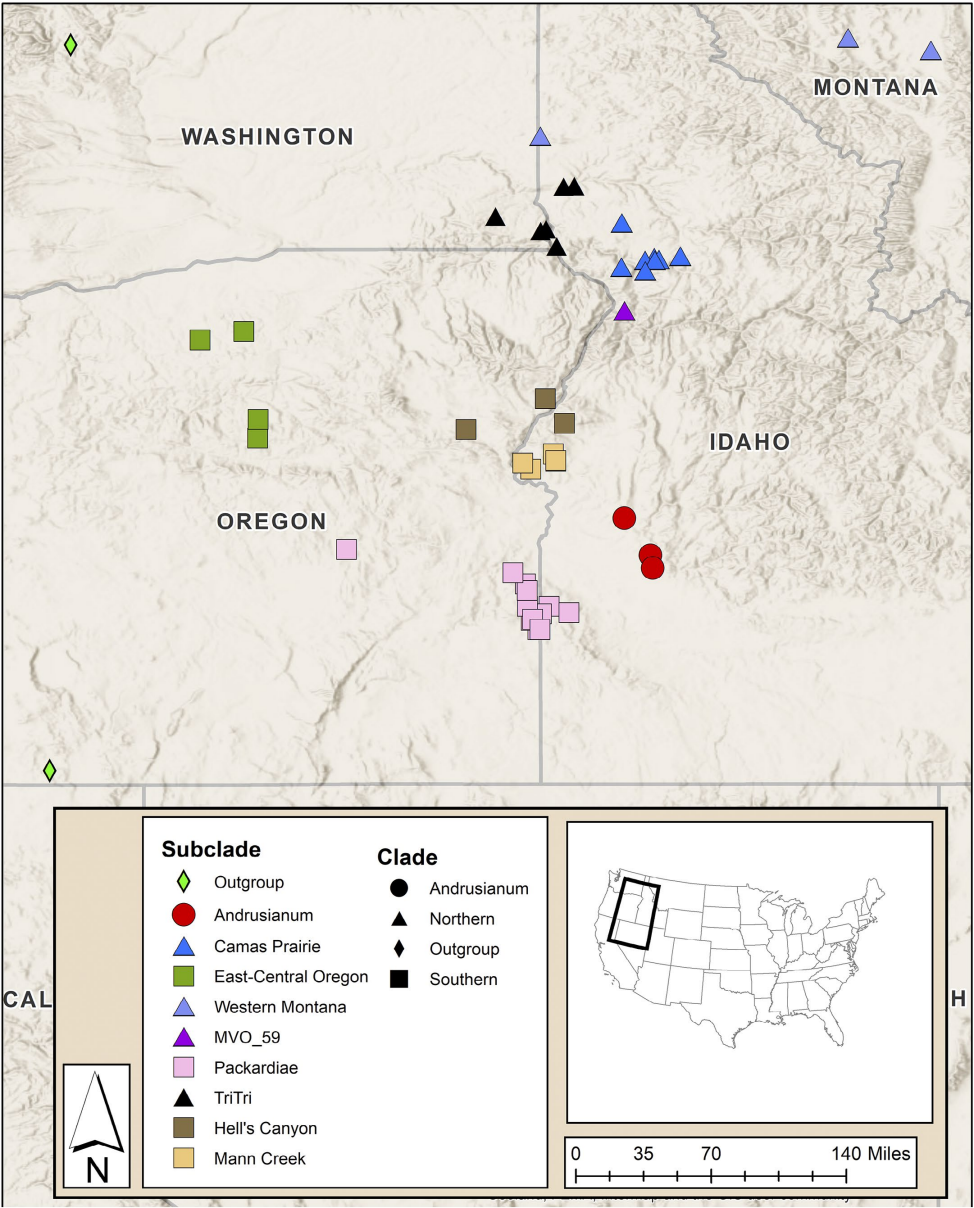
Map depicting locations of the samples and the associated subclades uncovered in the STACEY analysis. Outgroup samples (*Ottenlips 80*; *Smith 13048*) from the concatenated Bayesian inference and ASTRAL‐III analyses are included as light green diamonds. The STACEY clades are represented by shapes (circle = *L. andrusianum*, triangle = northern, square = southern). The STACEY subclades are color‐coded (pink = *L. packardiae*, yellow = Mann Creek, brown = Hell’s Canyon, green = East‐Central Oregon, blue = Camas Prairie, black = *L. triternatum* var. *triternatum*, gray‐blue = western Montana, and red = *L. andrusianum*).

Forty‐eight samples were chosen for molecular analysis (Fig. 2; Appendix S2), including 24 previously collected samples that had been preserved in silica gel and newly collected material from the 2017 and 2018 field seasons (Appendix S2). Populations that had been included by Smith et al. (2018a) were used to reconstruct the backbone of the broader *Lomatium triternatum* species complex, into which the newly collected individuals could be placed (Appendix S2). These new samples were included to expand the range of collections, decrease the geographic distance between previously collected individuals, and to sample a broader range of morphological intermediates spanning the narrow‐ and wide‐leaflet populations.

#### DNA extraction and shearing

DNA was extracted from silica‐dried leaf material using the Qiagen DNeasy plant minikit (Valencia, CA, USA) following the manufacturer’s recommended protocol, with slight modifications as described by Smith et al. (2018a) and George et al. (2014). DNA concentration was quantified using a Qubit 4 fluorometer with the dsDNA HS assay kit (Thermo Fisher Scientific, Waltham, MA, USA). A subset of 15 samples was randomly chosen for analysis on the Agilent 4200 TapeStation (Agilent Technologies, Santa Clara, CA, USA) using a genomic DNA tape to determine the molecular weight and quality of the samples. Output graphs were visually inspected for a peak around 20 kbp, indicating the genomic DNA was of high molecular weight and in need of shearing. Samples that were degraded did not need further shearing in the sonication step. DNA was sheared using a Covaris ME 220 Focused‐ultrasonicator (Covaris, Woburn, MA, USA) with 58 µL of input DNA per sample, for 65 s, with a peak power of 40 W, an average power of 4 W, a duty factor of 10%, and 1000 cycles per burst.

#### Library preparation

Libraries were prepared using the NEBNext Ultra II kit (New England BioLabs, Ipswich, MA, USA) according to the manufacturer’s recommendations with the following modifications: dual‐index (i5 and i7) primers were used for barcodes, and size selection was performed with 30 µL of SPRIselect beads (Beckman Coulter, Brea, CA, USA) for a target insert size of 400–500 bp. The Agilent 4200 TapeStation was used to check the fragment‐size distribution of the libraries; 2 µL of the high sensitivity buffer provided by the manufacturer and 2 µL of the sample were included in each well. Four D100 High Sensitivity (HS) tapes were placed in the machine. Since no D100 HS ladder was available, 1 µL of the D100 (non‐HS) ladder was diluted in 20 µL of neutral buffer. The machine was run according to the manufacturer’s specifications for the D100 HS kit. To quantify the concentration of double‐stranded DNA in each sample, a Quantus fluorometer (Promega, Madison, WI, USA) was used following the manufacturer’s recommended protocol.

#### Hybridization/target enrichment

All prepared libraries (100 ng of each DNA) were pooled into a single tube for one hybridization reaction using the Angiosperms353 bait kit manufactured by Arbor Biosciences (Ann Arbor, MI, USA). The hybridization reaction was performed following the MYbaits user manual version 3.02 (MYcroarray, 2016) with an incubation period of 24 hours in a Hybex Microsample Incubator (SciGene, Sunnyvale, CA, USA) at 65°C. The final product was amplified for 10 cycles, on‐bead, using a KAPA HiFi 2X HotStart ReadyMix PCR Kit (Roche, Basel, Switzerland). NebNext beads (New England BioLabs) at a DNA to bead ratio of 1:1 were used to clean the final PCR reaction. The final fragment size distribution and DNA concentration were quantified using the D100 HS kit on an Agilent 4200 TapeStation and a Quantus fluorometer, respectively.

#### Next‐generation sequencing

Sequencing was performed on an Illumina MiSeq L1000 (San Diego, CA, USA) with the MiSeq reagents kit v3 for 600 cycles, following a user‐determined final concentration of DNA to 16.5 pM in a 600 µL solution of the IlluminaHT1 reagent to sequence 2 × 300 paired‐end reads. DNA extractions were performed at Boise State University. All other laboratory work was performed at the Sackler Phylogenomics Laboratory at the Royal Botanic Gardens, Kew, UK.

### Bioinformatics workflow

Raw reads were demultiplexed with Illumina’s included software before downloading data from the MiSeq. Demultiplexed reads were checked for quality and adapter content with FastQC (Andrews, 2010). The paired‐end reads were combined and trimmed to remove leftover adapter fragments and low‐quality base pair calls (phred<33) using TRIMMOMATIC (Bolger et al., 2014) and the following parameters {‐phred33; ILLUMINACLIP:TruSeq3‐PE‐2.fa:2:30:7:2:true MAXINFO:40:0.7 CROP:290 MINLEN:36}. The HybPiper pipeline was used to assemble the genes targeted by the Angiosperms353 probe set. The pipeline maps individual reads to a target file using BWA (Li and Durbin, 2010) or BLAST (McGinnis et al., 2004) before a de novo assembly of each gene individually from the mapped reads with SPAdes (Bankevich et al., 2012) and annotates intron/exon boundaries sequences with Exonerate (Slater and Birney, 2005). The target files, provided by the developers of Angiosperms353 (https://github.com/mossmatters/Angiosperms353), contained reference genes from a wide range of angiosperm taxa ensuring its ability to map reads to each gene effectively across wide phylogenetic ranges. A benefit of this assembly method versus a solely reference‐based approach is the capture of the more‐variable flanking intron regions. The pipeline was run twice using different target files consisting of nucleotide (BWA method) or amino acid data (BLAST method). Theoretically, an amino acid target file will allow for greater variance in the mapped reads, resulting in assemblies with more depth and coverage. HybPiper was used to output both the exon and intron region(s) for each gene separately. Loci were aligned individually with MAFFT (Katoh and Standley, 2013) {parameters: ‐localpair ‐‐maxiterate 1000 ‐‐adjustdirectionaccurately} and alignments were cleaned to remove ambiguous regions using BMGE (Criscuolo and Gribaldo, 2010) with the default settings.

Basic alignment statistics, including sequence length, percentage missing data, percentage variable sites, and percentage parsimony informative sites were calculated with AMAS (Borowiec, 2016). Analyses were run both with all available data and separately with loci chosen for downstream analysis based on the following criteria adapted from Gernandt et al. (2018): all 48 samples present, less than 10% missing data, an alignment length of greater than or equal to 200 base pairs, less than 15% variable sites; and no paralog warnings generated by HybPiper. Loci criteria were applied using the custom script genepicker.R (https://github.com/ottenlipsmv/lom_pack_anom) on exons and introns, independently.

All scripts used in the assembly workflow including any detailed parameters unspecified in the methods can be found at https://github.com/ottenlipsmv/lom_pack_anom.

### Phylogenetic reconstruction

#### Species tree analyses

STACEY (Jones, 2014 [Preprint]) is a BEAST2 (Bouckaert et al., 2014) package that models the speciation process by incorporating coalescent theory and as such does not assume that individual gene trees are the same as the species tree (as assumed with MrBayes). This methodology is thus more realistic for groups that have undergone rapid and/or recent radiations and are expected to have a high degree of ILS. Outgroup samples [*Lomatium brevifolium* (J.M.Coult. & Rose) J.M.Coult. & Rose (*Smith 13048*) and *Lomatium thompsonii* (Mathias) Cronquist (*Ottenlips 80*)] were excluded from this analysis because preliminary STACEY runs suggested that the more distantly related outgroup taxa were drastically increasing computation time by causing MCMC mixing problems. Therefore, results from STACEY were rooted using *L. andrusianum* based on its phylogenetic position in previous studies (Smith et al., 2018a). Runs that included outgroup taxa had similar topologies to those presented here, but inadequate effective sample size (ESS) values. Five independent chains of STACEY were run for 5 billion generations, each with a collapse height set to 0.0001, a Yule‐death rate, and the JC69 molecular evolution model allowing STACEY to estimate the base‐pair substitution rate. The BEAST2 xml file with additional unspecified priors and MCMC parameters is available at https://github.com/ottenlipsmv/lom_pack_anom. LogCombiner (Rambaut and Drummond, 2013a) and Loganalyser (Bouckaert et al., 2014) were used to evaluate ESS values for all priors and parameters. A burn‐in of 50% was implemented for all log and tree files based on inspection of outputs from Loganalyser. Tree files were combined into a maximum clade credibility tree using LogCombiner and then annotated, visualized, and edited with TreeAnnotater (Rambaut and Drummond, 2013b), FigTree (manually re‐rooted using the *L. andrusianum* clade; Rambaut, 2007), and TreeGraph2 (collapsed nodes with <50% Bayesian posterior probabilities into polytomies; Stöver and Müller, 2010).

ASTRAL‐III (Zhang et al., 2017) is a species tree analysis program that measures gene tree discordance while estimating a species tree given a set of gene trees. ASTRAL‐III outputs three support values (posterior probabilities) for each node: one value for the main topology, a second for the probability that the clade belongs to the sister group, and a third value for the probability that the clade belongs to the outgroup (https://github.com/smirarab/ASTRAL/). Input gene trees were generated with RAxML (Stamatakis, 2014), stopping bootstrapping automatically using the auto‐MRE feature. The ASTRAL‐III analysis includes the outgroup samples excluded from the STACEY analysis.

#### Concatenated analysis

A Bayesian analysis was performed on a data set consisting of a concatenated alignment of the 54 introns filtered by the criteria identified above. Concatenation was performed by AMAS (Borowiec, 2016) and alignments by MAFFT (Katoh and Standley, 2013) {parameters: ‐localpair ‐maxiterate 1000}. A concatenated analysis assumes gene trees are identical to the overall species tree and thus does not account for ILS or hybridization. The model of molecular evolution (GTR+I+G) and base‐pair substitution rates were identified with Aikake information criterion (AIC) calculations in jModeltest2 (Darriba et al., 2012) on an unpartitioned data set and input as priors into MrBayes (Ronquist et al., 2012) with the following Monte Carlo, Markov chain (MCMC) parameters: two independent runs consisting of 20,000,000 generations, sampling every 1000 generations, a burn‐in of 25%, and four chains. *Lomatium brevifolium* (*Smith 13048*) and *L. thompsonii* (*Ottenlips 80*) were set as the outgroup taxa. Tracer (Rambaut and Drummond, 2003) was used to evaluate ESS values, burn‐in period, and convergence of other priors.

The bioinformatics pipeline and all evolutionary‐history reconstruction techniques with the exception of MrBayes were performed on a machine running Linux Ubuntu with 32 Intel Xeon Gold 6130 CPUs and 48 GB of RAM. MrBayes was run on the CIPRES Science Gateway (Miller et al., 2011).

### Soil, climatic, and morphological analyses

#### Soil data collection and analysis

Preliminary data (unpublished results) had indicated that soil types may be a factor in separating and delimiting species boundaries in the *Lomatium triternatum* complex. Therefore, soil data were collected for five replicate samples at each population site. Five soil compaction readings were taken with a pocket penetrometer (Forestry Suppliers, Jackson, MS, USA) in the four corners and at the center of each square meter quadrat. Approximately 200–500 g of soil was collected haphazardly from the upper horizons (ca. 4–12 cm below surface) at each replicate. Soil samples were mixed for each quadrat and were air dried inside a greenhouse at Boise State University. Dried soil samples were ground with a mortar and pestle, and gravels were separated from fines with a 2‐mm sieve to determine the gravel fraction. The hydrometer method (Bouyoucos, 1962) was used to determine the fines fraction. Due to calibration issues with the hydrometer, the fraction between sand and silt could not be determined. All soil particle analyses were performed at the soil laboratory in the Department of Geosciences at Boise State University. Approximately 150–200 g of dried, ground, and sieved soil was sent to the Soil and Forage Analysis lab at the University of Wisconsin‐Madison for routine and base saturation tests (soil pH, P, K, Ca, Mg, cation exchange capacity [CEC], organic matter [OM]), and additional soil tests (sulfate, soluble salts, N, nitrate, ammonia). Raw soil chemistry, compaction, and particle size data are provided in Appendix S3.

Soil characteristics were analyzed with a principal component analysis (PCA) to uncover distinct groupings that might correspond to monophyletic groups and/or distinct populations. Soil chemistry, compaction, and particle‐size data were centered and scaled and a PCA performed on the combined data set.

All data curation and analysis for ecological, morphological, and climatic variables was performed in R version 3.5.1 (R Core Team, 2018) and visualized using R packages ggplot2 (Wickham, 2016) and ggfortify (Tang et al., 2016). Principal components analyses were performed in base R.

#### Climatic data collection and analysis

Climate data were downloaded using the R package raster (Hijmans and van Etten, 2014) in the form of 19 BIOCLIM variables (Fick and Hijmans, 2017) at the 30 arc‐second resolution (~1 km^2^) from the UC‐Davis Biogeography webserver (http://biogeo.ucdavis.edu/data/climate/worldclim/). BIOCLIM variables were extracted for the GPS coordinates for each of the 48 individuals included in the molecular analysis using the extract function from the R package raster. The data were centered and scaled, and a PCA performed. Raw BIOCLIM variables for each collection are available as Appendix S4.

#### Morphological data collection and analysis

Both vegetative and reproductive characters were measured from herbarium specimens, including the samples used in the molecular analysis. Each measurement was replicated five times, and the means for each specimen were calculated. Leaflets and umbels that were completely visible with no overlap from other material on the herbarium sheet were chosen for measurements. The following reproductive traits were quantitively measured: ray length, mature fruit length, mature fruit width, mature pedicel length, primary umbel length, primary umbel width, and lateral rib wing width. Fruiting material was only available for 17 populations, but at least one population for each subclade identified in STACEY (see Results) was available (Appendix S5). A PCA was performed on the centered and scaled reproductive character means. Vegetative traits (leaflet width and length) were visualized separately by constructing a scatterplot with width on the *y*‐axis and length on the *x*‐axis. Preliminary analysis on additional vegetative characters (adapted from George et al., 2014) were uninformative, so only leaflet width and length were retained. Morphological data as averages are provided in Appendices S1 and S5 for vegetative and reproductive data, respectively.

## RESULTS

### Sample collection

Herbarium specimens and ecological data were collected for a total of 69 populations in flower between April and July of 2017, and 12 populations were revisited later in 2017 and in 2018 to obtain fruiting material. Notable collections included the type localities of *Lomatium packardiae* and *Lomatium triternatum* var. *triternatum*: *Ottenlips 20* and *Mansfield 16078*, respectively. Forty‐eight specimens that represented the morphological, ecological, and geographic variation of the subcomplex were chosen from material that was collected in the 2017 field season and previously collected samples from Smith et al. (2018a) for inclusion in the target‐enrichment NGS methods (Fig. 2).

### Assembly summary

Approximately 13% of the reads mapped back to the target loci. The average number of loci per sample with any mapped reads was 335. An average of 121 loci for each sample were at least 75% the target gene length, and 190 loci were at least 50% the target length. The average locus length was about 1021 bp.

Fifty‐four introns met the filtering criteria to be included in a downstream phylogenetic analysis (Appendix S6). Only 54 of the 353 loci sequenced were included in the downstream analysis due to strict filtering criteria (all 48 samples present, less than 10% missing data, an alignment length of greater than or equal to 200 bp, less than 15% variable sites; and no paralog warnings generated by HybPiper).

### Phylogenetic reconstruction

#### Species tree analyses

**FIGURE 3:**
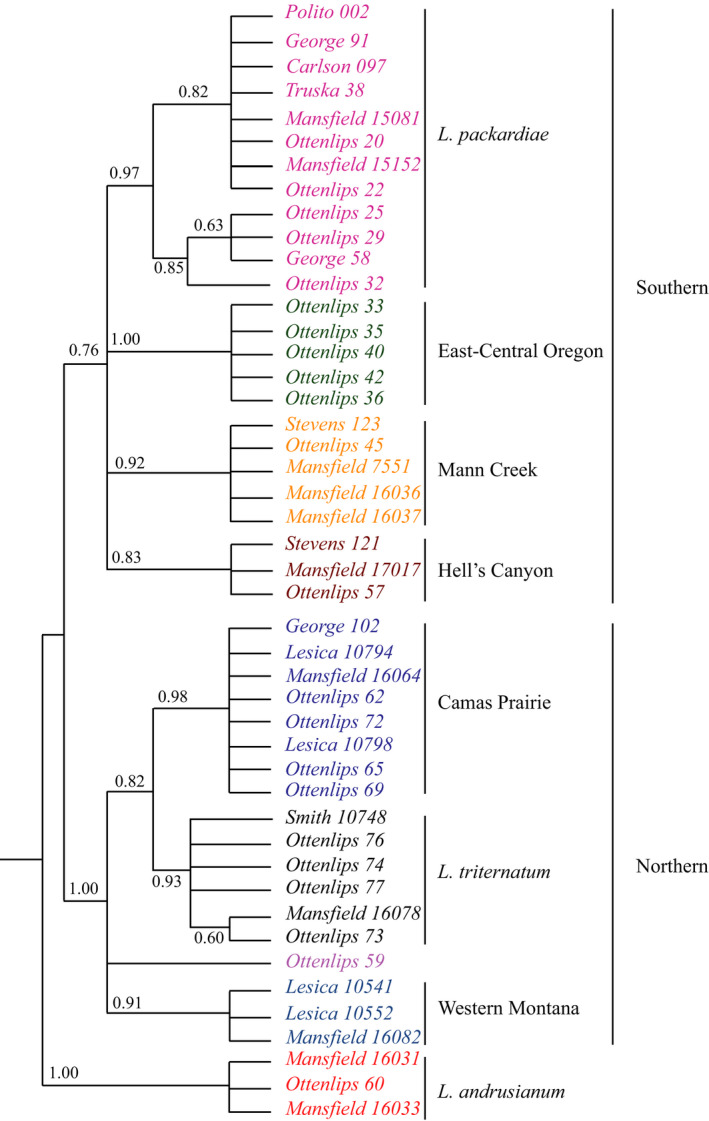
STACEY analysis of 54 introns. Colors correspond to clades described in the Discussion and the morphological and climatic analyses. Support values are posterior probabilities. Nodes with values under 0.50 are collapsed. The tree is manually rooted on *L. andrusianum*. The subclades are color‐coded (pink = *L. packardiae*, yellow = Mann Creek, brown = Hell’s Canyon, green = East‐Central Oregon, blue = Camas Prairie, black = *L. triternatum* var. *triternatum*, gray‐blue = western Montana, red = *L. andrusianum*).

The five combined runs of STACEY with a burn‐in of 50% for each run had ESS values of 631 for the posterior probability and 779 for the species‐tree coalescent model (https://github.com/ottenlipsmv/lom_pack_anom). The combined STACEY runs resulted in seven moderately to well‐supported subclades (posterior probabilities = 0.83 to 1.0) that agree with the geography and some of the traditionally recognized taxa within this species complex (Figs. 2 and 3). At a more inclusive scale, three larger clades are evident, splitting the species complex into northern and southern clades. The previously recognized *L*. *andrusianum* from the foothills northwest of Boise, Idaho was used to root this tree following the topology of Smith et al. (2018a).

**FIGURE 4:**
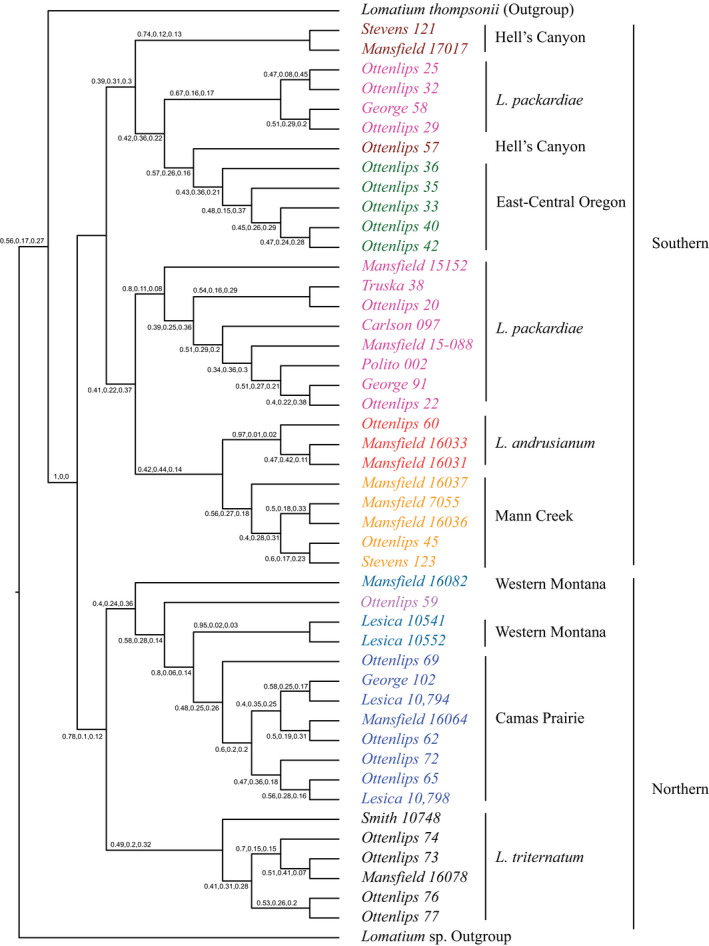
ASTRAL‐III species tree of 54 introns. The three support values on each branch are for the quad partition and show three posterior probabilities for each node: one value for the main topology, one value for probability the clade belongs to the sister group, and one value for the probability that the clade belongs to the outgroup. The STACEY subclades are color‐coded (pink = *L. packardiae*, yellow = Mann Creek, brown = Hell’s Canyon, green = East‐Central Oregon, blue = Camas Prairie, black = *L. triternatum* var. *triternatum*, gray‐blue = western Montana, red = *L. andrusianum*).

ASTRAL‐III analysis was performed on the full set of exons and introns recovered and on the filtered exons and introns to minimize the impact of missing data (Larridon et al., 2020). The topology of the full data analyses largely agrees with that of the filtered data, but with lower support, which is likely due to large amounts of missing data for the samples (Fig. 4; Appendix S7) and are not discussed further. The ASTRAL‐III species tree based on the filtered introns (Fig. 4) was to some degree congruent with the STACEY analysis, but with lower posterior probabilities for the main topology, suggesting a high degree of gene‐tree discordance. The main discrepancies occurred in the southern clade from the STACEY analysis; *L. packardiae* was not recovered as monophyletic, nor was the Hell's Canyon clade (Figs. 3, 4). Additionally, the placement of *L. andrusianum* was within the southern clade.

#### Concatenated analysis

The total length of the concatenated alignment of all introns was 55,152 characters, with 5.36% missing data, 18.3% variable sites, 6.7% parsimony informative, and a GC content of 33.4%. The two independent runs of MrBayes resulted in trees with identical topologies. All nodes were resolved with >0.99 posterior probabilities, except one with a support value of 0.64 (Appendix S8). Clades recovered in the concatenated analysis were mostly congruent with geographic separation and the species tree analyses with the following exceptions: two from Mann Creek (*Stevens 123*, *Stevens 121*), either of the Hell’s Canyon samples (*Mansfield 17017, Ottenlips 57*), one sample of *L. andrusianum* (*Mansfield 16031*), one sample from the Camas Prairie (*Ottenlips 69*), four samples of *L. packardiae* (*Ottenlips 25*, *Ottenlips 32*, *Ottenlips 20*, *George 58*), and two samples of *L. triternatum* (*Ottenlips 76*, *Ottenlips 77*) and one from western Montana (*Mansfield 16082*).

### Soil, climatic, and morphological analyses

Principal components 1 and 2 explained 24.3% and 20.9% of the variation in the soils data, respectively (Appendix S9). No clear clusters in the PCA agreed with the subclades distinguished by the STACEY analysis (Fig. 3). Two collections (*Ottenlips 69* and *Ottenlips 72*) are outliers that are both found near the Camas Prairie outside Grangeville, Idaho, with their placement in the PCA driven primarily by nitrate, total nitrogen, organic matter, and soluble salts content.

**FIGURE 5:**
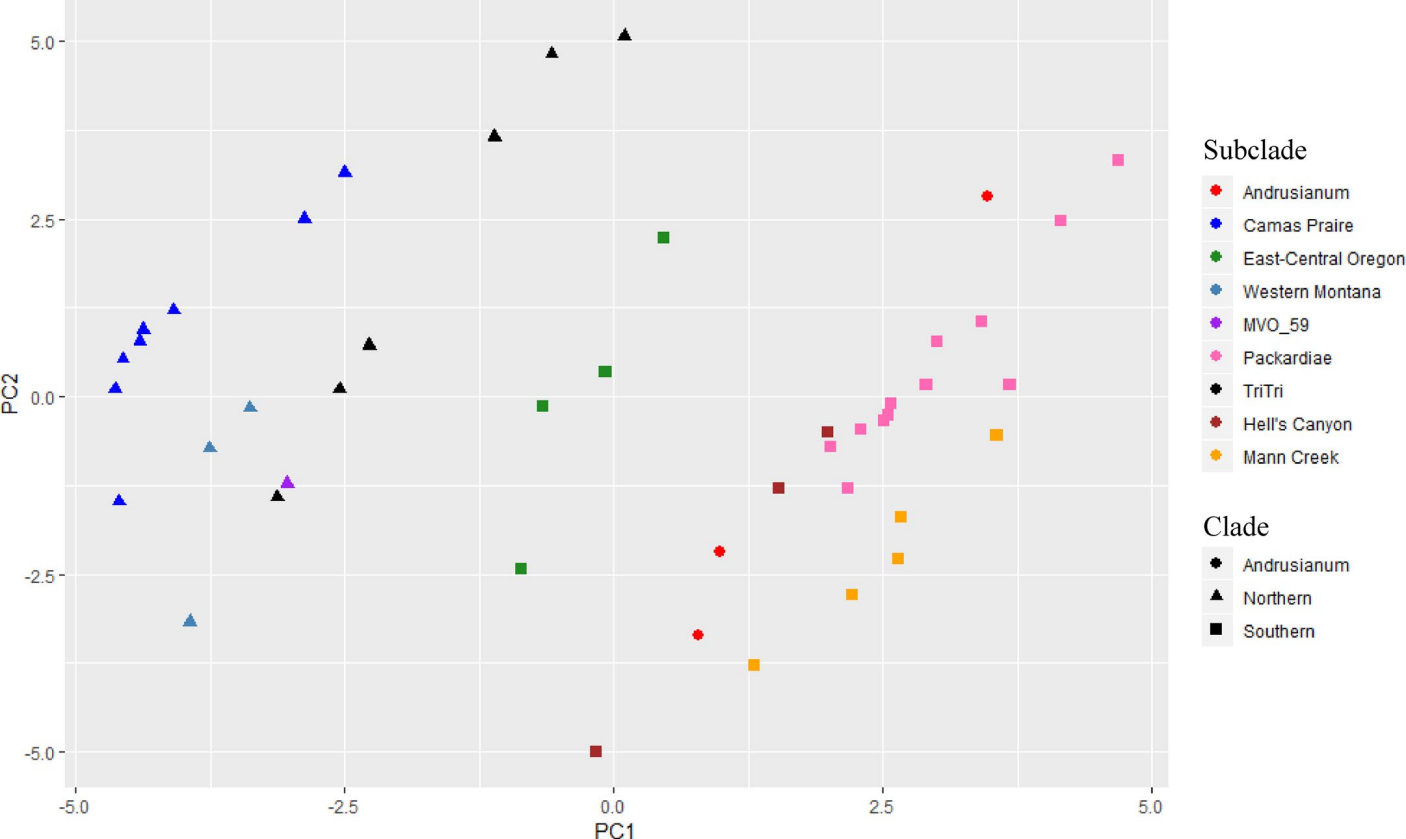
Principal component analysis on 19 centered and scaled bioclimatic variables extracted at 30 arc‐second resolution. The STACEY clades are represented by shapes (circle = *L. andrusianum*, triangle = northern, square = southern). The STACEY subclades are color‐coded (pink = *L. packardiae*, yellow = Mann Creek, brown = Hell’s Canyon, green = East‐Central Oregon, blue = Camas Prairie, black = *L. triternatum* var. *triternatum*, gray‐blue = western Montana, and red = *L. andrusianum*).

Principal components 1 and 2 explained 44.05% and 24.86% of the variation in the climatic data, respectively. Two clear clusters were evident in the analysis: the northern and southern clades, with some overlap in the East‐Central Oregon populations (Fig. 5).

Principal components 1 and 2 explained 41.82% and 24.86% of the variation in the fruit morphology data. The *Lomatium packardiae* populations cluster together, all sharing similarities in ray length and fruit size. Other previously recognized taxa, such as *L*. *andrusianum*, are not evident in our fruit morphology analysis (Appendix S10). Appendices S1, S5 contains the morphological data matrix as averages.

Most northern populations have longer and wider leaflets (1–13.4 mm average width; Appendix S1) than the southern populations (1–5 mm average width; Appendix S1), but there is substantial overlap in length and width, primarily in the clades from Washington County (Hell’s Canyon and Mann Creek; Appendix S1). Most populations from Camas Prairie are separated from all others in the scatterplot of leaflet width versus leaflet length distinguished by their longer and wider leaves (Appendix S11). Appendix S1 contains the average leaflet length and width for each specimen sampled.

## DISCUSSION

In the current study, we recovered clades that agree with described species (*Lomatium packardiae*) or geographic regions (Figs. 2, 3) that were not recovered in any prior molecular analysis (George et al., 2014; Smith et al., 2018a) or identifiable with keys based on morphological data (Hitchcock and Cronquist, 1973; Cronquist et al., 1997). Previous studies using Sanger‐based sequencing that have sampled far more individuals than were included here were sufficient to recover several clades (Smith et al., 2018a), but unable to resolve boundaries among accessions of *L. packardiae* and *L. anomalum*. In addition, our current results resolve taxonomic puzzles that Smith et al. (2018a) identified, such as the sister group relationship of *L. packardiae* (*Carlson 097*) with that of *L. triternatum* (*Mansfield 16078*). These two collections are separated geographically by nearly 200 linear miles and are morphologically distinct in terms of leaf width and fruit length. In our present analysis with the Angiosperms353 data, all analyses (Figs. 3, 4; Appendices S7, S8) have these two accessions widely separated and in the coalescent‐based analyses (Figs. 3, 4; Appendix S7) they are in agreement with geography and morphology; *Carlson 097* with narrow leaflets, is with *L. packardiae* in the southern clade and *Mansfield 16078* with wider leaflets is with other collections from near the type locality of *L. triternatum* in the northern clade.

Neither the concatenated data set analyzed using Bayesian inference of concatenated data, nor the ASTRAL‐III coalescent analysis of individual loci (using either all loci or a filtered set of loci to reduce missing data) recovered clades that are in full agreement with geography, morphology, or previous taxonomic concepts (Figs. 2, 4; Appendices S7, S8). Incomplete lineage sorting or interspecific hybridization could alter the results of the concatenated sequence data if a small number of loci with strong signal but incongruent with the species tree had a greater impact on the gene tree than the remaining loci, each with a weaker signal (Blom et al., 2017). The results from the Bayesian concatenated data set analysis differed from the STACEY analysis in that only the East‐Central Oregon clade (Fig. 3; Appendix S8) was monophyletic. It is unlikely that interspecific hybridization is the explanation here. Although previous phylogenetic studies using Sanger data did not always recover clades with strong support, there has been no detectable incongruence between plastid and nuclear ribosomal loci (Smith et al., 2018a), nor has there been any evidence of morphological intermediacy where species are sympatric. Thus, the discrepancy is most likely the result of ILS as also uncovered recently in *Cornus* (Thomas et al., 2021b). STACEY and ASTRAL‐III resolved phylogenetic relationships in the presence of ILS and thus are likely better indicators of species‐level phylogeny in this group.

The coalescent‐based analyses should be expected to recover similar estimates, but the results from ASTRAL‐III (Fig. 4) and STACEY (Fig. 3) did not fully agree. In the ASTRAL‐III analysis, *L. packardiae* and the Hell’s Canyon clade (the former strongly supported, posterior probability (pp) = 0.97, the latter less so, pp = 0.83 in STACEY; Fig. 3) were not recovered as monophyletic. The accession *Ottenlips 59* was unresolved in the STACEY tree (Fig. 3) and embedded in the western Montana grade in ASTRAL‐III (Fig. 4). Both analyses recovered the northern and southern clades, although neither clade is strongly supported with ASTRAL‐III and only the northern clade is strongly supported with STACEY. STACEY and ASTRAL‐III have different algorithms for handling ILS, and we suspect that the level of ILS relative to the strength of signal in each of the genes in our study group is the reason that clades in agreement with morphology and geography are recovered in STACEY and receive greater support. STACEY has been shown to be less sensitive to population size estimates compared to the software Bayesian Phylogenetics and Phylogeography (BPP; Yang and Rannala, 2010) and may explain why it performs better than ASTRAL‐III here (Barley et al., 2018).

The STACEY phylogeny resulting from the analysis of 54 introns was the only method to recover clades consistent with external, non‐molecular data (Figs. 2, 3, 5). Geography, and to some degree morphology and previously recognized taxonomy, mostly agree with phylogenetic position in the STACEY analysis. For example, STACEY recovered *Lomatium packardiae* as monophyletic and supported. Although both northern and southern clades were also recovered with ASTRAL‐III, only STACEY recovered structure within these clades that corresponds to small geographic areas such as western Montana and Camas Prairie (Fig. 3). Therefore, we will focus the remainder of our discussion on this analysis because it is likely that the discrepancies from other analyses are due to ILS.

The STACEY phylogeny revealed a major geographic split between northern and southern populations; this split was also observed in ASTRAL‐III, but not in the concatenated Bayesian nuclear analysis. Within the larger clades, STACEY uncovered seven less inclusive subclades that correspond to geography (Figs. 2, 3) and to some degree morphology and previously recognized taxonomy, such as the recovery of *L. packardiae* as both monophyletic (Fig. 3) and morphologically distinct from other accessions in the PCA of reproductive characters (Appendix S10). Neither the ASTRAL‐III, nor concatenated nuclear Bayesian tree recovered these subclades with as much support as the STACEY topology (Figs. 3, 4; Appendix S8).

### Efficacy of introns and coalescent theory in shallow‐scale phylogenetic analysis

Johnson et al. (2016, 2018) have discussed the theoretical practicality of using the highly variable introns at the population or species‐complex levels, but this investigation into the evolutionary history of the *Lomatium triternatum* complex is the first study to our knowledge to demonstrate the efficacy of using only the flanking intronic regions from a target‐enrichment approach at shallow species‐complex‐level scales. Previous studies of the *Lomatium triternatum* complex failed to resolve relationships among the *Lomatium anomalum/packardiae* subcomplex and recovered unusual results regarding the placement of some individuals (Smith et al., 2018a). The use of introns from the Angiosperms353 probe set allowed us to recover structure within this subcomplex that agreed with geography, previously recognized species boundaries, and to a lesser extent, morphology. Previous attempts most likely failed because of low levels of sequence variation and substantial amounts of ILS in this group. A recent paper investigated the utility of the Angiosperms353 exons to resolve phylogenetic estimates at the species level in *Cyperus* (Larridon et al., 2020). This study obtained well‐resolved and strongly supported estimates using exons, but also recovered largely congruent topologies using concatenated and coalescent approaches, likely indicating that ILS is not a significant issue in this group of *Cyperus* (despite being a relatively shallow radiation) when compared to the *Lomatium anomalum/L. packardiae* complex. In contrast, Thomas et al. (2021b) uncovered likely ILS in the Hydrostachyaceae and *Cornus*. Larridon et al. (2020) also compared results obtained with Cyperaceae‐specific baits and found that they were not better at resolving relationships than those acquired with the Angiosperms353 probe set, thus indicating that use of the Angiosperms353 probe set can be as productive a method without the time and cost associated with the development of taxon‐specific baits. Similarly, Thomas et al. (2021a) used both introns and exons from the Angiosperms353 probe set to better resolve phylogenetic relationships in *Veronica* sect. *Hebe* (Plantaginaceae), a radiation estimated at 5–10 million years ago.

Most studies at low taxonomic scales use presence/absence data, including restriction site associated DNA sequencing (RAD‐seq) derived single nucleotide polymorphisms (SNPs) or microsatellites. Examples include investigations into speciation with gene flow in lake whitefishes with RAD‐seq data (Gagnaire et al., 2013) and the creation of a genetic linkage map using microsatellites in the *Mimulus guttatus* species complex (Fishman et al., 2001). However, presence/absence data may not fully account for ILS due to the lack of full sequence data for intact genes.

Coalescent‐based methodologies have been shown to outperform concatenated approaches in recovering accurate evolutionary relationships, especially at shallow time scales where ILS may be more common (Degnan and Rosenberg, 2009; Larridon et al., 2020). Additionally, because they assume that some genes have coalesced within lineages that are not the most recent common ancestor (Baum and Shaw, 1995), coalescent models can more accurately represent the biological reality of the speciation process. They are also better fits for the genealogical species concept when compared to concatenation because they allow for the possibility that individual gene trees may have different evolutionary histories, which in turn may differ from the species tree. Our sampling scheme of multiple individuals allows STACEY to estimate more accurately the population size parameter of the coalescent‐based model. Additionally, no previous studies have demonstrated the use of STACEY with such a large number (54) of loci derived from NGS data due to intense computational demand (Barley et al., 2018; Tomasello, 2018; Matos‐Maraví et al., 2019; Wagner et al., 2019; Femenias et al., 2020). Introns contain enough variation to be useful at shallower levels but have the added benefit of being easily incorporated into coalescent‐based models.

Angiosperms353 allowed us to use low‐copy nuclear loci, both introns and exons, without having to develop taxon‐specific baits to better resolve the relationships in this complex. This ultimately permitted use of coalescent based analyses that would not have been fully possible with SNPs and would not have resolved relationships in this complex as well. The Angiosperms353 probe set proved to be relatively easy to use. The lead author spent a little over 2 weeks learning the methodology and had the raw data needed for this study ready shortly after that for bioinformatics processing. Likewise, the HybPiper software (Johnson et al., 2016) facilitated the data assembly. The use of loci that are common among multiple studies will allow future integration of individual studies and projects more readily. Many of the authors on this publication are already collaborating on projects that investigate the PENA clade more broadly as well as species boundaries in two other complexes in this group. We will be able to integrate the data from this study into those and make full use of outgroups among the PENA clade to fully test species boundaries in other complexes. Additionally, we will be able to broaden our potential outgroup sampling as others such as Clarkson et al. (2021) sample more broadly across Apiaceae, and reciprocally the data generated by our work can be incorporated into theirs.

### Climate and morphological variation

A comparison of climate, morphological, and phylogenetic data indicates that some morphological variation corresponds to variation in climate, suggesting that the morphology may be potentially plastic and responding to ecological and environmental parameters. The environment of the northern clade has cooler and wetter temperatures than the southern clade (Fig. 5; Appendix S4). Most populations found in the north have wide leaflets, resembling the traditionally recognized taxon *Lomatium anomalum*. The southern clade’s environment is hotter and drier (Fig. 5; Appendix S4). Populations found in the south generally have shorter and narrower leaflets and many have been previously treated as *L*. *packardiae* (Figs. 1, 2). Because environmental factors are correlated with geography, it is difficult to ascertain fully whether the morphological variation found in the northern and southern clades represent a phenotypic response to environmental conditions or is genetically derived. However, not all populations in the north share the wide *anomalum*‐like leaflet morphology. Notably, *Mansfield 16082* from western Montana and *Ottenlips 74* from the *L. triternatum* population both have an average leaflet width of 2 mm (Appendix S1). In general, individuals from the subclade corresponding to *L. triternatum* var. *triternatum*, especially those found in Asotin County, Washington, have narrow leaflets more similar to *L*. *packardiae*. Also, individuals from Washington County, which are members of the southern clade, have wider leaflets in contrast to the more representative narrow leaflets found in members of the southern clade.

### Species boundaries

The recovery of geographically separated, and to a lesser extent, morphologically distinguishable subclades with nuclear coalescent‐based approaches, but not with the concatenated analysis, indicates that ILS has prevented previous recognition of distinct clades within this subcomplex. While interspecific hybridization is a possible explanation for the discrepancy between concatenated and coalescent approaches, no previous study has detected evidence of hybridization (Smith et al., 2018a), nor has any morphological evidence emerged that would suggest hybridization. A possible explanation for the high level of ILS is that the clades recovered with the STACEY analysis are the result of incipient speciation, which is cryptic due to the lack of any consistently reliable diagnostic traits besides geography. Until we have sampled more widely within this species complex using the methodology presented in this paper, we hesitate to recognize any new species within the *L. anomalum/L. packardiae* subcomplex (southern Clade). The seven subclades recovered from the STACEY analysis may be distinct or incipient species. Several clades that are strongly supported in this analysis correspond to existing names (*L. packardiae*, *L. andrusianum*) or to clades that were suspected as distinct based on geographic distribution and leaf morphology (East‐Central Oregon, Camas Prairie). These may all be valid species under the genealogical species concept (Baum and Shaw, 1995; monophyletic and at the reticulate divergent boundary) but would then leave clades that receive less support (*L. triternatum* var. *triternatum*, western Montana, Hell’s Canyon, Mann Creek) outside of a species or as a poorly supported monophyletic species. The entirety of the northern clade is maximally supported, so we currently propose that the name *L. triternatum* s.s. be applied to all of the samples in this clade (Camas Prairie, *Ottenlips 59,* and western Montana) in addition to the clade that most closely agrees to the type locality of *L. triternatum* (labeled as *L. triternatum* var. *triternatum*). The southern clade is not strongly supported (pp = 0.76), but two of its subclades are (*L. packardiae* and East‐Central Oregon). The southern clade creates a more difficult situation, especially given that the individuals from Mann Creek do not fit morphologically within *L. packardiae* (or *L. andrusianum*; see Stevens et al., 2018).

The four southern subclades are also part of a polytomy; however morphologically and geographically, *L. packardiae* and the East‐Central Oregon populations are more similar, and likewise, the Mann Creek and Hell’s Canyon populations are more similar. In an earlier study, Smith et al. (2018a, see their fig. 3) found that according to leaflet morphology, *L. packardiae* and the Mann Creek/Hell’s Canyon separated into two forms, Form A and Form B, respectively. In addition, fruit length was found to average >10.0 mm for *L. packardiae* (Form A of Smith et al., 2018a, their fig. 3) and the East‐Central Oregon population (sp. 2 of Smith et al., 2018a), whereas fruit length averaged >14.0 mm for the Mann Creek and Hell’s Canyon populations (Form B of Smith et al., 2018a). In addition, according to Smith et al. (2018a, p. 226), sp. 2 (East Central Oregon) “has been confused with *L. packardiae* in the past.” Therefore, we propose to group East‐Central Oregon with *L. packardiae* and to recognize the remaining two subclades (Mann Creek and Hell’s Canyon) as *L. anomalum*. The geographic distribution and morphology of these two subclades largely matches that of the type locality of *L. anomalum*, and so they will retain this name. Despite our current inability to recover these clades (*L. packardiae*/East‐Central Oregon and Hel’s Canyon/Mann Creek) as monophyletic, we cannot preclude that they are not and recognizing these as two species is our current best, but not perfect, taxonomic solution.

## CONCLUSIONS

The evolutionary history and morphological variation of the *L. packardiae/L. anomalum* subcomplex is likely a combination of periodic populations newly founded by few individuals, ILS caused by incipient or recent speciation, phenotypic plasticity in response to ecological parameters, and geographic structuring in the nuclear genome due to limited gene flow from pollinators with short travel distances (Cane et al., 2020) and no clear mechanism for seed dispersal. Although we base our conclusions on some known studies of pollinators (Cane et al., 2020) and the data we recovered here that indicates the presence of ILS in geographically structured populations, we are limited in that studies have yet to be done on founding populations and phenotypic plasticity. To our knowledge, there are no population genetic studies that might verify that populations of *Lomatium* are founded by few individuals. Likewise, to our knowledge, there are no studies that have investigated the dispersal of *Lomatium* fruits and certainly not the species considered here. However, the fruits do not seem to have any clear means of dispersal other than gravity (D. H. Mansfield, J. F. Smith, personal observations). We did find that the northern populations tend to have wider leaflets and occupy wetter habitats, but we cannot yet discern if the wider leaves are the result of adaptation or phenotypic plasticity. Concatenated nuclear, coalescent‐based, morphometric, and ecological analyses provide evidence to help understand these patterns of evolutionary history and morphological variation. Denser sampling of morphological and environmental parameters could help to untangle the role of ecologically determined plasticity, especially in the Camas Prairie and *L. triternatum* var. *triternatum* clades. Further investigations into potential hybridization are also necessary, although based on previous studies and morphology, there is no current indication that hybridization is or has occured in this group. Bayesian coalescent‐based phylogenetic reconstruction techniques performed on introns extracted from target‐enrichment NGS protocols were crucial to understanding these recalcitrant taxa, and this technique could be more broadly applied in recent and ongoing radiations at the species complex and population levels across a wide variety of angiosperm groups. We add evidence to a growing body of literature (Ješovnik et al., 2017; Coates et al., 2018; Jacobs et al., 2018; Villaverde et al., 2018; Pie et al., 2019; Loureiro et al., 2020; Sproul et al., 2020) that demonstrates the value of targeted sequencing techniques to resolve species and population level phylogenetic relationships and conclude that introns as well as exons derived from the Angiosperms353 probe set can be especially useful to achieve resolution at this taxonomic level.

## AUTHOR CONTRIBUTIONS

**Michael V. Ottenlips:** Conceptualization (equal); Data curation (equal); Formal analysis (equal); Investigation (equal); Methodology (equal); Software (equal); Writing – original draft (equal). **Donald H. Mansfield:** Conceptualization (equal); Methodology (equal); Project administration (equal); Resources (equal); Supervision (equal); Writing – review & editing (equal). **Sven Buerki:** Formal analysis (equal); Software (equal); Supervision (equal); Writing – review & editing (equal). **Mary Ann E. Feist:** Formal analysis (equal); Investigation (equal); Validation (equal); Writing – review & editing (equal). **Stephen R. Downie:** Validation (equal); Writing – review & editing (equal). **Steven Dodsworth:** Funding acquisition (equal); Methodology (equal); Resources (equal); Writing – review & editing (equal). **Félix Forest:** Funding acquisition (equal); Methodology (equal); Project administration (equal); Resources (equal); Writing – review & editing (equal). **Gregory M. Plunkett:** Validation (equal); Writing – review & editing (equal). **James F. Smith:** Conceptualization (equal); Data curation (equal); Formal analysis (equal); Funding acquisition (equal); Project administration (equal); Resources (equal); Supervision (equal); Writing – review & editing (equal).

M.V.O., J.F.S., D.H.M., and M.A.E.F. designed the project; M.V.O., J.F.S., and D.H.M. conducted fieldwork; M.V.O., S.D., and F.F. facilitated and conducted the Angiosperms353 data collection; M.V.O. and S.B. conducted bioinformatics analyses; and all authors contributed to writing and editing of the final manuscript.

## Supporting information

**APPENDIX S1**. The mean of five replicate measurements for leaflet width and length taken from herbarium collections.

**APPENDIX S2**. Voucher information and which specimens were available for each analysis.

**APPENDIX S3**. Raw soil chemistry and physical properties data.

**APPENDIX S4**. Climatic variables for each collection.

**APPENDIX S5**. Summary of reproductive character measurements.

**APPENDIX S6**. Assembly statistics of the 54 introns calculated with AMAS.

**APPENDIX S7**. ASTRAL‐III species tree based on exons and introns.

**APPENDIX S8**. Majority‐rule Bayesian inference consensus tree of concatenated data.

**APPENDIX S9**. Principal component analysis of centered and scaled soil data.

**APPENDIX S10**. Principal component analysis of eight centered and scaled means of reproductive characters.

**APPENDIX S11**. Scatterplot depicting the average leaflet lengths and widths organized by the clades uncovered in the STACEY analysis.

**APPENDIX S12**. GenBank accessions for 54 introns used in this study.

## Data Availability

Sequences are deposited in GenBank and listed in Appendix S12. Custom scripts used in the analyses are available at https://github.com/ottenlipsmv/lom_pack_anom.
