# Supplementary material for: Resolving species boundaries in a recent radiation with the Angiosperms353 probe set: the Lomatium packardiae/L. anomalum clade of the L. triternatum (Apiaceae) complex
Source: Am J Bot. 2021 Jun 8;108(7):1217–33. doi: 10.1002/ajb2.1676 (PMC8362113; doi:10.1002/ajb2.1676)
Supplement: Supplementary file 1 — APPENDIX S1. The mean of five replicate measurements for leaflet width and length taken from herbarium collections. [file AJB2-108-1217-s009.docx]

## Ottenlips et al.—American Journal of Botany 2021—Appendix S1

## Appendix S1. The mean of five replicate measurements for leaflet width and length taken from herbarium collections. All measurements in mm. Further information on accessions and vouchers are in Appendix S2.

| Accession | Collection abbreviation | STACEY Clade/Subclade | Length | Width |
| --- | --- | --- | --- | --- |
| *Mansfield 15088* | DM_15088 | Southern/*L. packardiae* | 8.2 | 1 |
| *Mansfield 16031* | DM_16031 | *L. andrusianum* | 18 | 1.8 |
| *Mansfield 16033* | DM_16033 | *L. andrusianum* | 10.8 | 2.2 |
| *Mansfield 16036* | DM_16036 | Southern/Mann Creek | 25.2 | 3.4 |
| *Mansfield 16037* | DM_16037 | Southern/Mann Creek | 17.6 | 2.8 |
| *Mansfield 16064* | DM_16064 | Northern/Camas Prairie | 41 | 13.4 |
| *Mansfield 16078* | DM_16078 | Northern/*L. triternatum* | 29.6 | 3.4 |
| *Mansfield 16082* | DM_16082 | Northern/Western Montana | 59.8 | 2 |
| *Mansfield 17017* | DM_17017 | Southern/Hell’s Canyon | 16.6 | 1.9 |
| *Mansfield 7055* | DM_7-55 | Southern/Mann Creek | 23 | 4.2 |
| *George 102* | EG_102 | Northern/Camas Prairie | 36.4 | 10.8 |
| *George 58* | EG_58 | Southern/*L. packardiae* | 14.6 | 1.4 |
| *George 91* | EG_91 | Southern/*L. packardiae* | 14 | 1.2 |
| *Carlson 97* | KC_097 | Southern/*L. packardiae* | 10.8 | 1.4 |
| *Stevens 121* | MS_121 | Southern/Hell’s Canyon | 19 | 2.4 |
| *Stevens 123* | MS_123 | Southern/Mann Creek | 33.4 | 4.8 |
| *Ottenlips 32* | MVO_32 | Southern/*L. packardiae* | 12.4 | 1.3 |
| *Ottenlips 76* | MVO_76 | Northern/*L. triternatum* | 28.2 | 2.5 |
| *Ottenlips 77* | MVO_77 | Northern/*L. triternatum* | 52.2 | 2.2 |
| *Lesica 10541* | PL_10541 | Northern/Western Montana | 48.2 | 4.6 |
| *Lesica 10552* | PL_10552 | Northern/Western Montana | 51.6 | 5 |
| *Lesica 10794* | PL_10794 | Northern/Camas Prairie | 36.2 | 9.4 |
| *Lesica 10978* | PL_10978 | Northern/Camas Prairie | 43.8 | 10.2 |
| *Ottenlips 65* | MVO_65 | Northern/Camas Prairie | 30 | 5 |
| *Ottenlips 42* | MVO_42 | Southern/East-Central Oregon | 20 | 2 |
| *Ottenlips 40* | MVO_40 | Southern/East-Central Oregon | 20 | 1 |
| *Ottenlips 74* | MVO_74 | Northern/*L. triternatum* | 40 | 1 |
| *Ottenlips 25* | MVO_25 | Southern/*L. packardiae* | 4 | 1 |
| *Ottenlips 69* | MVO_69 | Northern/Camas Prairie | 32 | 4 |
| *Ottenlips 60* | MVO_60 | *L. andrusianum* | 20 | 3 |
| *Ottenlips 57* | MVO_57 | Southern/Hell’s Canyon | 60 | 5 |
| *Ottenlips 35* | MVO_35 | Southern/East-Central Oregon | 15 | 2 |
| *Ottenlips 33* | MVO_33 | Southern/East-Central Oregon | 15 | 1 |
| *Ottenlips 29* | MVO_29 | Southern/*L. packardiae* | 40 | 3 |
| *Ottenlips 22* | MVO_22 | Southern/*L. packardiae* | 20 | 1 |
| *Ottenlips 59* | MVO_59 | Northern/NA | 55 | 5 |
| *Ottenlips 45* | MVO_45 | Southern/Mann Creek | 25 | 4 |
| *Ottenlips 62* | MVO_62 | Northern/Camas Prairie | 30 | 10 |
| *Ottenlips 20* | MVO_20 | Southern/*L. packardiae* | 25 | 3 |
| *Ottenlips 36* | MVO_36 | Southern/East-Central Oregon | 25 | 1 |
| *Ottenlips 73* | MVO_73 | Northern/*L. triternatum* | 50 | 3 |
| *Ottenlips 72* | MVO_72 | Northern/Camas Prairie | 43 | 8 |
