# Supplementary material for: Resolving species boundaries in a recent radiation with the Angiosperms353 probe set: the Lomatium packardiae/L. anomalum clade of the L. triternatum (Apiaceae) complex
Source: Am J Bot. 2021 Jun 8;108(7):1217–33. doi: 10.1002/ajb2.1676 (PMC8362113; doi:10.1002/ajb2.1676)
Supplement: Supplementary file 2 — APPENDIX S2. Voucher information and which specimens were available for each analysis. [file AJB2-108-1217-s003.docx]

## Ottenlips et al.—American Journal of Botany 2021—Appendix S2

## Appendix S2. Voucher information and which specimens were available for each analysis.

| Accession | Herbarium acronym | Collection abbreviation | Location | STACEY Clade/Subclade | Vegetative | Sanger | Environmental | Soil | NGS |
| --- | --- | --- | --- | --- | --- | --- | --- | --- | --- |
| *Ottenlips 80* | SRP | MVO_80 | Chelan County, Washington | *L. thompsonii* | Y | N | Y | Y | Y |
| *Mansfield 16031* | SRP | DM_16031 | Ada County, Idaho | *L. andrusianum* | N | Y | Y | N | Y |
| *Mansfield 16033* | SRP | DM_16033 | Ada County. Idaho | *L. andrusianum* | N | Y | Y | N | Y |
| *Mansfield 16078* | CIC | DM_16078 | Nez Perce County, Idaho | Northern/*L. triternatum* | N | Y | Y | N | Y |
| *Carlson 97* | CIC | KC_097 | Owyhee County, Idaho | Southern/*L. packardiae* | N | Y | Y | N | Y |
| *Lesica 10552* | CIC | PL_10552 | Lake County, Montana | Northern/Western Montana | N | Y | Y | N | Y |
| *Lesica 10541* | CIC | PL_10541 | Sanders County, Montana | Northern/Western Montana | N | Y | Y | N | Y |
| *Mansfield 16082* | CIC | DM_16082 | Latah County, Idaho | Northern/Western Montana | N | Y | Y | N | Y |
| *George 102* | CIC | EG_102 | Idaho County, Idaho | Northern/Camas Prairie | N | Y | Y | N | Y |
| *Lesica 10978* | CIC | PL_10,798 | Idaho County, Idaho | Northern/Camas Prairie | N | Y | Y | N | Y |
| *Lesica 10794* | CIC | PL_10,794 | Idaho County, Idaho | Northern/Camas Prairie | N | Y | Y | N | Y |
| *Ottenlips 65* | SRP | MVO_65 | Idaho County, Idaho | Northern/Camas Prairie | Y | N | Y | Y | Y |
| *Mansfield 16036* | CIC | DM_16036 | Washington County, Idaho | Southern/Mann Creek | N | Y | Y | N | Y |
| *Mansfield 07055* | CIC | DM_7055 | Washington County, Idaho | Southern/Mann Creek | N | Y | Y | N | Y |
| *George 58* | CIC | EG_58 | Malheur County, Oregon | Southern/*L. packardiae* | N | Y | Y | N | Y |
| *Smith 13048* | SRP | JFS_13048 | Lake County, Oregon | *L. brevifolium* | N | Y | Y | N | Y |
| *Ottenlips 42* | SRP | MVO_42 | Morrow County, Oregon | Southern/East-Central Oregon | Y | N | Y | Y | Y |
| *Ottenlips 40* | SRP | MVO_40 | Morrow County, Oregon | Southern/East-Central Oregon | Y | N | Y | Y | Y |
| *Truska 38* | CIC | Truska_38 | Malheur County, Oregon | Southern/*L. packardiae* | N | Y | Y | N | Y |
| *Mansfield 15088* | CIC | DM_15-088 | Owyhee County, Idaho | Southern/*L. packardiae* | N | Y | Y | N | Y |
| *Polito 002* | CIC | LP_002 | Owyhee County, Idaho | Southern/*L. packardiae* | N | Y | Y | N | Y |
| *George 91* | CIC | EG_91 | Malheur County, Oregon | Southern/*L. packardiae* | N | Y | Y | N | Y |
| *Ottenlips 74* | SRP | MVO_74 | Asotin County, Washington | Northern/*L. triternatum* | Y | N | Y | Y | Y |
| *Ottenlips 76* | SRP | MVO_76 | Asotin County, Washington | Northern/*L. triternatum* | N | N | Y | Y | Y |
| *Ottenlips 77* | SRP | MVO_77 | Asotin County, Washington | Northern/*L. triternatum* | N | N | Y | Y | Y |
| *Ottenlips 25* | SRP | MVO_25 | Malheur County, Oregon | Southern/*L. packardiae* | Y | N | Y | Y | Y |
| *Ottenlips 69* | SRP | MVO_69 | Idaho County, Idaho | Northern/Camas Prairie | Y | N | Y | Y | Y |
| *Mansfield 15-152* | CIC | DM_15152 | Owyhee County, Idaho | Southern/*L. packardiae* | N | Y | Y | N | Y |
| *Smith 10748* | SRP | JFS_10748 | Nez Perce County, Idaho | Northern/*L. triternatum* | N | Y | Y | N | Y |
| *Mansfield 16037* | CIC | DM_16037 | Washington County, Idaho | Southern/Mann Creek | N | Y | Y | Y | Y |
| *Ottenlips 32* | SRP | MVO_32 | Harney County, Oregon | Southern/*L. packardiae* | N | N | Y | Y | Y |
| *Ottenlips 60* | SRP | MVO_60 | Gem County, Idaho | *L. andrusianum* | Y | N | Y | Y | Y |
| *Ottenlips 57* | SRP | MVO_57 | Washington County, Idaho | Southern/Hell’s Canyon | Y | N | Y | Y | Y |
| *Ottenlips 35* | SRP | MVO_35 | Grant County, Oregon | Southern/East-Central Oregon | Y | N | Y | Y | Y |
| *Ottenlips 33* | SRP | MVO_33 | Grant County, Oregon | Southern/East-Central Oregon | Y | N | Y | Y | Y |
| *Ottenlips 29* | SRP | MVO_29 | Malheur County, Oregon | Southern/*L. packardiae* | Y | N | Y | Y | Y |
| *Ottenlips 22* | SRP | MVO_22 | Malheur County, Oregon | Southern/*L. packardiae* | Y | N | Y | Y | Y |
| *Ottenlips 59* | SRP | MVO_59 | Idaho County, Idaho | Northern/NA | Y | N | Y | Y | Y |
| *Mansfield 16064* | CIC | DM_16064 | Idaho County, Idaho | Northern/Camas Prairie | N | Y | Y | N | Y |
| *Ottenlips 45* | SRP | MVO_45 | Washington County, Idaho | Southern/Mann Creek | Y | N | Y | Y | Y |
| *Ottenlips 62* | SRP | MVO_62 | Idaho County, Idaho | Northern/Camas Prairie | Y | N | Y | Y | Y |
| *Ottenlips 20* | SRP | MVO_20 | Owyhee County, Idaho | Southern/*L. packardiae* | Y | N | Y | Y | Y |
| *Stevens 121* | CIC | MS_121 | Baker County, Oregon | Southern/Hell’s Canyon | N | Y | Y | Y | Y |
| *Stevens 123* | CIC | MS_123 | Washington County, Oregon | Southern/Mann Creek | N | Y | Y | Y | Y |
| *Ottenlips 36* | SRP | MVO_36 | Grant County, Oregon | Southern/East-Central Oregon | Y | N | Y | Y | Y |
| *Mansfield 17017* | CIC | DM_17017 | Baker County, Oregon | Southern/Hell’s Canyon | N | Y | Y | N | Y |
| *Ottenlips 73* | SRP | MVO_73 | Nez Perce County, Idaho | Northern/*L. triternatum* | Y | N | Y | Y | Y |
| *Ottenlips 72* | SRP | MVO_72 | Nez Perce County, Idaho | Northern/Camas Prairie | Y | N | Y | Y | Y |
