# Supplementary material for: Resolving species boundaries in a recent radiation with the Angiosperms353 probe set: the Lomatium packardiae/L. anomalum clade of the L. triternatum (Apiaceae) complex
Source: Am J Bot. 2021 Jun 8;108(7):1217–33. doi: 10.1002/ajb2.1676 (PMC8362113; doi:10.1002/ajb2.1676)
Supplement: Supplementary file 3 — APPENDIX S3. Raw soil chemistry and physical properties data. [file AJB2-108-1217-s004.docx]

## Ottenlips et al.—American Journal of Botany 2021—Appendix S3

## Appendix S3. Raw soil chemistry and physical properties data.

| Accession | Collection abbreviation | STACEY Clade/Subclade | Soil_pH | P_ppm | Bray1_K_ppm | Ca_ppm | Mg_ppm | Na_ppm | CEC_sum | OM | Sulfate_ppm | Soluble_salts | N | nitrate | amonia | gravel | clay | sand_and_silt |
| --- | --- | --- | --- | --- | --- | --- | --- | --- | --- | --- | --- | --- | --- | --- | --- | --- | --- | --- |
| *Ottenlips 20* | MVO_20 | Southern/*L. packardiae* | 6.9 | 48 | 653 | 3247 | 401 | 89 | 18 | 3.80161 | 4.1 | 0.35 | 1755.77 | 19.9 | 7 | 60.7895 | 37.9658 | 62.0342 |
| *Ottenlips 22* | MVO_22 | Southern/*L. packardiae* | 7.1 | 36 | 405 | 3499 | 1044 | 164 | 18 | 3.86001 | 2.7 | 0.22 | 1336.79 | 3.1 | 6.8 | 67.4234 | 48.0144 | 51.9856 |
| *Ottenlips 25* | MVO_25 | Southern/*L. packardiae* | 7.6 | 16 | 336 | 2759 | 621 | 231 | 15 | 2.2705 | 1.4 | 0.29 | 843.468 | 3.7 | 3.6 | 30.5889 | 34 | 66 |
| *Ottenlips 29* | MVO_29 | Southern/*L. packardiae* | 7.1 | 66 | 545 | 2907 | 723 | 48 | 16 | 3.73674 | 2.3 | 0.21 | 1801.1 | 10.9 | 4 | 16.1484 | 40.008 | 59.992 |
| *Ottenlips 32* | MVO_32 | Southern/*L. packardiae* | 7.1 | 27 | 192 | 4153 | 630 | 88 | 20 | 2.18951 | 0.8 | 0.28 | 672.357 | 3.1 | 4.1 | 1.83552 | 68.0068 | 31.9932 |
| *Ottenlips 33* | MVO_33 | Southern/East-Central Oregon Northern/Western Montana | 6.7 | 21 | 172 | 3089 | 1058 | 31 | 16 | 3.96606 | 2.8 | 0.21 | 1790.05 | 8.5 | 7.1 | 10.1006 | 35.9928 | 64.0072 |
| *Ottenlips 35* | MVO_35 | Southern/East-Central Oregon Northern/Western Montana | 7.1 | 20 | 140 | 3227 | 1092 | 34 | 14 | 2.79122 | 2.9 | 0.25 | 1041.03 | 6.4 | 9.1 | 40.0951 | 37.9962 | 62.0038 |
| *Ottenlips 36* | MVO_36 | Southern/East-Central Oregon | 6.9 | 13 | 171 | 2334 | 1047 | 35 | 10 | 3.17588 | 1.7 | 0.15 | 1534.95 | 6.4 | 7.6 | 38.7924 | 28.9913 | 71.0087 |
| *Ottenlips 40* | MVO_40 | Southern/East-Central Oregon | 7.8 | 34 | 214 | 6624 | 437 | 24 | 34 | 3.66231 | 19.6 | 0.27 | 1589.21 | 4.5 | 7.4 | 19.499 | 40.9918 | 59.0082 |
| *Ottenlips 42* | MVO_42 | Southern/East-Central Oregon | 6.9 | 24 | 258 | 2610 | 649 | 45 | 13 | 3.15615 | 4.2 | 0.21 | 1437.26 | 9.9 | 7.9 | 38.9585 | 33.0066 | 66.9934 |
| *Ottenlips 45* | MVO_45 | Southern/Mann Creek | 7 | 54 | 258 | 3519 | 1162 | 43 | 20 | 2.99615 | 1.5 | 0.23 | 812.527 | 2 | 11.4 | 29.4146 | 57.9826 | 42.0174 |
| *Ottenlips 57* | MVO_57 | Southern/Hell’s Canyon | 6.7 | 29 | 244 | 2484 | 496 | 220 | 16 | 3.40369 | 2.8 | 0.14 | 1441.36 | 2.4 | 11.6 | 41.5451 | 24.985 | 75.015 |
| *Ottenlips 59* | MVO_59 | Northern/NA | 6.4 | 44 | 183 | 2127 | 276 | 15 | 12 | 4.47077 | 5 | 0.12 | 2352.36 | 10.8 | 6.3 | 39.6089 | 25.9844 | 74.0156 |
| *Ottenlips 60* | MVO_60 | *L. andrusianum* Southern/Mann Creek | 6.5 | 45 | 235 | 3408 | 856 | 29 | 20 | 4.95427 | 4.2 | 0.25 | 2077.94 | 2.5 | 12.3 | 30.7358 | 47.976 | 52.024 |
| *Ottenlips 62* | MVO_62 | Northern/Camas Prairie | 6.3 | 7 | 83 | 3280 | 1049 | 41 | 17 | 2.97109 | 1.3 | 0.2 | 1216.15 | 16.2 | 3.3 | 26.803 | 32.3069 | 67.6931 |
| *Ottenlips 65* | MVO_65 | Northern/Camas Prairie | 5.9 | 16 | 153 | 2339 | 601 | 48 | 15 | 4.54464 | 5.4 | 0.2 | 2126.82 | 26.6 | 4.8 | 41.3407 | 37.9507 | 62.0493 |
| *Ottenlips 69* | MVO_69 | Northern/Camas Prairie | 5.8 | 5 | 261 | 2759 | 809 | 19 | 20 | 7.71278 | 5.5 | 0.6 | 4292.75 | 153 | 3.4 | 20.7059 | 31.1495 | 68.8505 |
| *Ottenlips 72* | MVO_72 | Northern/Camas Prairie | 6.3 | 35 | 335 | 3958 | 407 | 28 | 25 | 6.24873 | 4.2 | 0.31 | 2941.42 | 36.4 | 44.3 | 40.8583 | 43.9341 | 56.0659 |
| *Ottenlips 73* | MVO_73 | Northern/*L. triternatum* | 6.9 | 24 | 355 | 7158 | 816 | 19 | 34 | 3.57964 | 2.5 | 0.25 | 1498.02 | 11.3 | 44.4 | 44.0935 | 43 | 57 |
| *Ottenlips 74* | MVO_74 | Northern/*L. triternatum* | 6.9 | 20 | 209 | 4180 | 671 | 17 | 19 | 2.58565 | 1.4 | 0.15 | 1246.68 | 8.4 | 2.9 | 32.9555 | 27.8996 | 72.1004 |
| *Ottenlips 76* | MVO_76 | Northern/*L. triternatum* | 6.7 | 47 | 363 | 3861 | 497 | 17 | 22 | 4.35281 | 2 | 0.12 | 2325.18 | 4.5 | 6.1 | 29.5254 | 38.1986 | 61.8014 |
| *Ottenlips 77* | MVO_77 | Northern/*L. triternatum* | 6 | 24 | 184 | 1893 | 392 | 19 | 13 | 3.39382 | 2.4 | 0.16 | 1920.26 | 21.4 | 3 | 0.12565 | 33.8949 | 66.1051 |
| *Stevens 121* | MS_121 | Southern/Hell’s Canyon | 7 | 22 | 156 | 2731 | 1076 | 40.4 | 14.4531 | 1.8 | 2.9 | 0.33 | 796 | 2.8 | 14.5 | 23.6749 | 22.1343 | 77.8657 |
| *Mansfield 16037* | DM_16037 | Southern/Mann Creek | 6.6 | 123 | 612 | 2843 | 807 | 40.4 | 19.2827 | 4.6 | 4.4 | 0.43 | 2515.8 | 4.5 | 12.6 | 20.8075 | 25.3416 | 74.6584 |
