# Supplementary material for: Resolving species boundaries in a recent radiation with the Angiosperms353 probe set: the Lomatium packardiae/L. anomalum clade of the L. triternatum (Apiaceae) complex
Source: Am J Bot. 2021 Jun 8;108(7):1217–33. doi: 10.1002/ajb2.1676 (PMC8362113; doi:10.1002/ajb2.1676)
Supplement: Supplementary file 4 — APPENDIX S4. Climatic variables for each collection. [file AJB2-108-1217-s005.docx]

## Ottenlips et al.—American Journal of Botany 2021—Appendix S4

## Appendix S4. Climatic variables for each collection. Codes correspond to the variable in the key: bio1_12 = Annual Mean Tempature (°C); bio2_12= Mean Diurnal Range (°C); bio3_12 = Isothermality (°C); bio5_12 = Max Temperature of the Warmest Month (°C); bio6_12 = Min Temperature of the Coldest Month (°C); bio7_12 = Temperature Annual Range (°C); bio8_12 = Mean Temperature of the Wettest Quarter (°C); bio9_12 = Mean Temperature of the Driest Quarter (°C); bio11_12 = Mean Temperature of the Warmest Quarter (°C); bio12_12 = Mean Temperature of the Coldest Quarter (°C); bio13_12 = Annual Precipitation (mm); bio14_12 = Precipitation of the Wettest Month (mm); bio16_12 = Precipitation of the Driest Month (mm); bio19_12 = Precipitation of the Wettest Quarter (mm).

| Accession | Collection abbreviation | STACEY Clade/Subclade |  | bio1_12 | bio2_12 | bio3_12 | bio4_12 | bio5_12 | bio6_12 | bio7_12 | bio8_12 | bio9_12 | bio10_12 | bio11_12 | bio12_12 | bio13_12 | bio14_12 | bio15_12 | bio16_12 | bio17_12 | bio18_12 | bio19_12 |
| --- | --- | --- | --- | --- | --- | --- | --- | --- | --- | --- | --- | --- | --- | --- | --- | --- | --- | --- | --- | --- | --- | --- |
| *Ottenlips 80* | MVO_80 | *L. thompsonii* |  | 95 | 139 | 36 | 8336 | 308 | -68 | 376 | -7 | 195 | 201 | -14 | 448 | 88 | 8 | 72 | 241 | 36 | 37 | 220 |
| *Mansfield 16031* | DM_16031 | *L. andrusianum* |  | 76 | 149 | 38 | 8149 | 301 | -88 | 389 | -18 | 178 | 183 | -27 | 486 | 70 | 11 | 46 | 199 | 50 | 57 | 188 |
| *Mansfield 16033* | DM_16033 | *L. andrusianum* |  | 106 | 140 | 36 | 8346 | 327 | -56 | 383 | 7 | 209 | 215 | -1 | 330 | 42 | 8 | 38 | 121 | 37 | 43 | 111 |
| *Mansfield 16078* | DM_16078 | Northern/*L. triternatum* |  | 116 | 129 | 37 | 7620 | 321 | -26 | 347 | 25 | 211 | 215 | 21 | 445 | 48 | 19 | 25 | 143 | 67 | 80 | 132 |
| *Carlson 97* | KC_097 | Southern/*L. packardiae* |  | 72 | 159 | 40 | 7731 | 297 | -91 | 388 | -17 | 169 | 175 | -24 | 354 | 40 | 11 | 31 | 120 | 46 | 59 | 112 |
| *Lesica 10552* | PL_10552 | Northern/Western Montana |  | 72 | 126 | 35 | 7914 | 277 | -79 | 356 | 112 | 26 | 176 | -28 | 439 | 59 | 25 | 28 | 150 | 88 | 124 | 97 |
| *Lesica 10541* | PL_10541 | Northern/Western Montana |  | 48 | 136 | 37 | 7708 | 261 | -104 | 365 | -42 | 143 | 149 | -48 | 575 | 65 | 30 | 22 | 182 | 110 | 128 | 172 |
| *Mansfield 16082* | DM_16082 | Northern/Western Montana |  | 77 | 125 | 37 | 6893 | 275 | -57 | 332 | -5 | 164 | 166 | -10 | 614 | 80 | 21 | 37 | 234 | 77 | 90 | 217 |
| *George 102* | EG_102 | Northern/Camas Prairie |  | 77 | 132 | 38 | 7085 | 278 | -63 | 341 | 106 | 167 | 172 | -9 | 586 | 77 | 28 | 29 | 205 | 103 | 125 | 125 |
| *Lesica 10978* | PL_10978 | Northern/Camas Prairie |  | 74 | 134 | 38 | 7095 | 276 | -68 | 344 | 102 | 164 | 169 | -13 | 600 | 77 | 29 | 27 | 207 | 105 | 128 | 130 |
| *Lesica 10794* | PL_10794 | Northern/Camas Prairie |  | 102 | 137 | 38 | 7592 | 315 | -43 | 358 | 96 | 197 | 203 | 8 | 600 | 71 | 26 | 26 | 195 | 97 | 116 | 140 |
| *Ottenlips 65* | MVO_65 | Northern/Camas Prairie |  | 79 | 132 | 38 | 7151 | 280 | -62 | 342 | 108 | 169 | 174 | -9 | 569 | 74 | 28 | 28 | 198 | 101 | 123 | 121 |
| *Mansfield 16036* | DM_16036 | Southern/Mann Creek |  | 90 | 151 | 36 | 8948 | 321 | -90 | 411 | -18 | 198 | 206 | -27 | 361 | 53 | 9 | 43 | 149 | 41 | 50 | 139 |
| *Mansfield 7055* | DM_7055 | Southern/Mann Creek |  | 82 | 155 | 37 | 8877 | 315 | -98 | 413 | -24 | 188 | 196 | -36 | 433 | 65 | 10 | 47 | 184 | 45 | 55 | 173 |
| *George 58* | EG_58 | Southern/*L. packardiae* |  | 102 | 157 | 39 | 8121 | 330 | -65 | 395 | 4 | 200 | 208 | -2 | 244 | 26 | 8 | 27 | 76 | 36 | 46 | 70 |
| *Smith 13048* | JFS_13048 | *L. brevifolium* |  | 73 | 143 | 41 | 6668 | 273 | -68 | 341 | -4 | 158 | 161 | -10 | 442 | 56 | 13 | 36 | 163 | 54 | 63 | 145 |
| *Ottenlips 42* | MVO_42 | Southern/East-Central Oregon |  | 88 | 140 | 41 | 6651 | 286 | -50 | 336 | 12 | 174 | 176 | 7 | 373 | 47 | 11 | 34 | 133 | 45 | 55 | 118 |
| *Ottenlips 40* | MVO_40 | Southern/East-Central Oregon |  | 75 | 163 | 41 | 7714 | 303 | -89 | 392 | -14 | 172 | 178 | -20 | 346 | 40 | 12 | 30 | 117 | 46 | 59 | 108 |
| *Truska 38* | Truska_38 | Southern/*L. packardiae* |  | 73 | 164 | 41 | 7701 | 301 | -90 | 391 | -15 | 170 | 176 | -22 | 346 | 39 | 12 | 30 | 115 | 46 | 59 | 107 |
| *Mansfield 15088* | DM_15088 | Southern/*L. packardiae* |  | 80 | 162 | 41 | 7679 | 305 | -83 | 388 | -10 | 175 | 182 | -17 | 315 | 34 | 11 | 28 | 100 | 43 | 56 | 93 |
| *Polito 002* | LP_002 | Southern/*L. packardiae* |  | 82 | 166 | 42 | 7673 | 310 | -82 | 392 | -9 | 176 | 183 | -15 | 301 | 32 | 11 | 27 | 92 | 43 | 55 | 85 |
| *George 91* | EG_91 | Southern/*L. packardiae* |  | 68 | 133 | 39 | 6974 | 269 | -71 | 340 | -13 | 157 | 159 | -19 | 587 | 72 | 20 | 34 | 213 | 77 | 91 | 196 |
| *Ottenlips 74* | MVO_74 | Northern/*L. triternatum* |  | 77 | 131 | 38 | 7156 | 279 | -64 | 343 | -7 | 168 | 170 | -12 | 522 | 55 | 22 | 25 | 163 | 81 | 97 | 148 |
| *Ottenlips 76* | MVO_76 | Northern/*L. triternatum* |  | 73 | 132 | 38 | 7136 | 275 | -69 | 344 | -11 | 163 | 166 | -17 | 537 | 57 | 22 | 25 | 170 | 83 | 99 | 155 |
| *Ottenlips 77* | MVO_77 | Northern/*L. triternatum* |  | 94 | 160 | 40 | 7939 | 321 | -71 | 392 | -1 | 190 | 199 | -7 | 264 | 28 | 9 | 27 | 82 | 38 | 50 | 76 |
| *Ottenlips 25* | MVO_25 | Southern/*L. packardiae* |  | 88 | 132 | 38 | 7290 | 292 | -53 | 345 | 118 | 181 | 185 | -1 | 519 | 66 | 25 | 26 | 177 | 91 | 111 | 114 |
| *Ottenlips 69* | MVO_69 | Northern/Camas Prairie |  | 70 | 156 | 40 | 7735 | 293 | -91 | 384 | -18 | 168 | 174 | -26 | 363 | 42 | 11 | 32 | 126 | 46 | 59 | 117 |
| *Mansfield 15152* | DM_15152 | Southern/*L. packardiae* |  | 105 | 131 | 37 | 7515 | 311 | -37 | 348 | 137 | 200 | 204 | 12 | 420 | 48 | 19 | 23 | 130 | 70 | 84 | 108 |
| *Smith 10748* | JFS_10748 | Northern/*L. triternatum* |  | 80 | 152 | 37 | 8773 | 309 | -98 | 407 | -25 | 186 | 193 | -35 | 389 | 57 | 11 | 43 | 159 | 46 | 57 | 149 |
| *Mansfield 16037* | DM_16037 | Southern/Mann Creek |  | 80 | 164 | 41 | 7988 | 308 | -92 | 400 | -15 | 178 | 185 | -22 | 283 | 36 | 11 | 32 | 102 | 41 | 50 | 92 |
| *Ottenlips 32* | MVO_32 | Southern/*L. packardiae* |  | 70 | 155 | 39 | 8235 | 297 | -100 | 397 | -27 | 170 | 177 | -37 | 513 | 72 | 12 | 45 | 210 | 54 | 63 | 198 |
| *Ottenlips 60* | MVO_60 | *L. andrusianum* |  | 58 | 155 | 38 | 8397 | 287 | -116 | 403 | -39 | 160 | 167 | -52 | 547 | 79 | 15 | 43 | 222 | 62 | 76 | 212 |
| *Ottenlips 57* | MVO_57 | Southern/Hell’s Canyon |  | 72 | 157 | 44 | 6524 | 281 | -72 | 353 | -3 | 154 | 159 | -9 | 406 | 47 | 16 | 28 | 137 | 61 | 73 | 119 |
| *Ottenlips 35* | MVO_35 | Southern/East-Central Oregon |  | 53 | 159 | 43 | 6688 | 264 | -98 | 362 | -23 | 138 | 142 | -30 | 443 | 55 | 17 | 31 | 157 | 65 | 78 | 138 |
| *Ottenlips 33* | MVO_33 | Southern/East-Central Oregon |  | 67 | 161 | 41 | 7822 | 294 | -98 | 392 | -24 | 164 | 170 | -32 | 360 | 43 | 12 | 31 | 125 | 48 | 61 | 116 |
| *Ottenlips 29* | MVO_29 | Southern/*L. packardiae* |  | 76 | 164 | 41 | 7686 | 305 | -87 | 392 | -12 | 173 | 179 | -19 | 330 | 37 | 11 | 30 | 109 | 44 | 56 | 101 |
| *Ottenlips 22* | MVO_22 | Southern/*L. packardiae* |  | 63 | 146 | 39 | 7404 | 275 | -91 | 366 | 94 | 156 | 160 | -30 | 583 | 64 | 24 | 25 | 178 | 91 | 112 | 159 |
| *Ottenlips 59* | MVO_59 | Northern |  | 76 | 133 | 38 | 7107 | 277 | -65 | 342 | 105 | 166 | 171 | -11 | 589 | 77 | 28 | 28 | 206 | 103 | 126 | 126 |
| *Mansfield 16064* | DM_16064 | Northern/Camas Prairie |  | 68 | 154 | 37 | 8664 | 299 | -109 | 408 | -34 | 173 | 180 | -46 | 480 | 71 | 12 | 45 | 200 | 52 | 64 | 190 |
| *Ottenlips 45* | MVO_45 | Southern/Mann Creek |  | 59 | 141 | 39 | 7125 | 264 | -89 | 353 | 87 | 149 | 153 | -29 | 621 | 74 | 28 | 25 | 201 | 104 | 126 | 154 |
| *Ottenlips 62* | MVO_62 | Northern/Camas Prairie |  | 74 | 163 | 41 | 7721 | 302 | -90 | 392 | -15 | 171 | 177 | -22 | 353 | 41 | 12 | 31 | 120 | 46 | 59 | 111 |
| *Ottenlips 20* | MVO_20 | Southern/*L. packardiae* |  | 87 | 154 | 38 | 8471 | 313 | -84 | 397 | -14 | 189 | 195 | -25 | 470 | 67 | 13 | 43 | 193 | 54 | 66 | 179 |
| *Stevens 121* | MS_121 | Southern/Hell’s Canyon |  | 78 | 154 | 37 | 8818 | 310 | -102 | 412 | -27 | 184 | 191 | -39 | 450 | 67 | 11 | 47 | 190 | 47 | 57 | 180 |
| *Stevens 123* | MS_123 | Southern/Mann Creek |  | 72 | 157 | 44 | 6524 | 281 | -72 | 353 | -3 | 154 | 159 | -9 | 406 | 47 | 16 | 28 | 137 | 61 | 73 | 119 |
| *Ottenlips 36* | MVO_36 | Southern/East-Central Oregon |  | 74 | 152 | 39 | 8140 | 295 | -93 | 388 | -24 | 174 | 178 | -32 | 337 | 42 | 14 | 29 | 118 | 53 | 68 | 108 |
| *Mansfield 17017* | DM_17017 | Southern/Hell’s Canyon |  | 102 | 125 | 36 | 7360 | 303 | -37 | 340 | 15 | 195 | 198 | 10 | 474 | 51 | 20 | 25 | 152 | 72 | 86 | 139 |
| *Ottenlips 73* | MVO_73 | Northern/*L. triternatum* |  | 68 | 129 | 37 | 7060 | 268 | -72 | 340 | 97 | 158 | 162 | -19 | 597 | 71 | 27 | 24 | 190 | 101 | 119 | 148 |
| *Ottenlips 72* | MVO_72 | Northern/Camas Prairie |  | 67 | 158 | 44 | 6682 | 275 | -81 | 356 | -7 | 154 | 155 | -14 | 436 | 58 | 11 | 40 | 164 | 46 | 58 | 154 |
