# Supplementary material for: Resolving species boundaries in a recent radiation with the Angiosperms353 probe set: the Lomatium packardiae/L. anomalum clade of the L. triternatum (Apiaceae) complex
Source: Am J Bot. 2021 Jun 8;108(7):1217–33. doi: 10.1002/ajb2.1676 (PMC8362113; doi:10.1002/ajb2.1676)
Supplement: Supplementary file 5 — APPENDIX S5. Summary of reproductive character measurements. [file AJB2-108-1217-s007.docx]

## Ottenlips et al.—American Journal of Botany 2021—Appendix S5

## Appendix S5. Summary of reproductive character measurements. Each cell is the mean of five replicate measures.

| mmAccession | Collection/abbreviation | STACEY Clade/Subclade | Ray length cm | Mature Fruit length mm | Mature Fruit Width mm | Mature Pedicel Length mm | Length of primary umbel cm | Width of primary umbel cm | Wing width mm | Fruit Length mm |
| --- | --- | --- | --- | --- | --- | --- | --- | --- | --- | --- |
| *Mansfield 16031* | DM_16031 | *L. andrusianum* | 8.1 | 12.6 | 4.8 | 4.4 | 7 | 8 | 1 | 4 |
| *Mansfield 16033* | DM_16033 | *L. andrusianum* | 10.6 | 10.6 | 4.2 | 4.2 | 13.5 | 12.5 | 1 | 4.5 |
| *Mansfield 16078* | DM_16078 | Northern/*L. triternatum* | 7.1 | 14.8 | 6 | 8.8 | 10 | 14 | 1 | 5.5 |
| *Mansfield 16082* | DM_16082 | Northern/Western Montana | 7.6 | 9.2 | 4.6 | 7.4 | 8.5 | 9 | 1 | 4 |
| *Mansfield 16036* | DM_16036 | Southern/Mann Creek | 3.8 | 18.4 | 6.4 | 4.8 | 7 | 6 | 2 | 6 |
| *Mansfield 7055* | MVO_42 | Southern/Mann Creek | 6.2 | 11.2 | 6 | 7 | 9 | 13 | 2 | 6 |
| *Mansfield 15088* | DM_15088 | Southern/*L. packardiae* | 5.4 | 7.8 | 4.2 | 4.6 | 8 | 9 | 0.75 | 3 |
| *George 91* | EG_91 | Southern/*L. packardiae* | 5.7 | 9.4 | 4 | 5.2 | 11 | 4 | 1 | 5 |
| *Ottenlips 25* | MVO_25 | Southern/*L. packardiae* | 5.4 | 7.6 | 3.6 | 4.8 | 7 | 6 | 1 | 3 |
| *Mansfield 15152* | DM_15152 | Southern/*L. packardiae* | 6.6 | 9 | 4 | 4.2 | 8 | 9 | 0.75 | 3 |
| *Mansfield 16037* | DM_16037 | Southern/Mann Creek | 8.6 | 14.8 | 5.7 | 6.6 | 11 | 12.5 | 1 | 4.5 |
| *Mansfield 16064* | DM_16064 | Northern/Camas Prairie | 11.8 | 10.6 | 4.6 | 4.4 | 17 | 15 | 1.25 | 6 |
| *Ottenlips 20* | MVO_20 | Southern/*L. packardiae* | 6.8 | 6.4 | 3 | 4.8 | 8 | 10 | 0.5 | 4 |
| *Stevens 121* | MS_121 | Southern/Hell’s Canyon | 10.2 | 15.4 | 4.2 | 8 | 13 | 7 | 1 | 4 |
| *Stevens 123* | MS_123 | Southern/Mann Creek | 6.9 | 13.2 | 4.6 | 8 | 8 | 8 | 1 | 4 |
| *Ottenlips 73* | MVO_73 | Northern/*L. triternatum* | 6.8 | 8.2 | 3.8 | 6 | 9 | 10 | 1 | 4 |
