# Supplementary material for: Resolving species boundaries in a recent radiation with the Angiosperms353 probe set: the Lomatium packardiae/L. anomalum clade of the L. triternatum (Apiaceae) complex
Source: Am J Bot. 2021 Jun 8;108(7):1217–33. doi: 10.1002/ajb2.1676 (PMC8362113; doi:10.1002/ajb2.1676)
Supplement: Supplementary file 6 — APPENDIX S6. Assembly statistics of the 54 introns calculated with AMAS. [file AJB2-108-1217-s011.docx]

## Ottenlips et al.—American Journal of Botany 2021—Appendix S6

Appendix S6. Assembly statistics of the 54 introns. Gene number refers to the unique identifier associated with the Angiosperm 353 bait kit target file as calculated in AMAS.

| Gene number | Alignment length | Missing percent | Percent parsimony informative | Percent variable sites | GC content (percentage) |
| --- | --- | --- | --- | --- | --- |
| 4848 | 729 | 6.076 | 2.5 | 15.2 | 36.3 |
| 4932 | 717 | 3.879 | 2.4 | 8.2 | 31.6 |
| 4992 | 1083 | 5.711 | 5.1 | 15.1 | 32.3 |
| 5116 | 1304 | 6.538 | 19.2 | 23.4 | 32.6 |
| 5271 | 550 | 3.545 | 2.7 | 9.6 | 32.6 |
| 5326 | 439 | 7.422 | 7.5 | 20 | 36 |
| 5333 | 272 | 3.676 | 9.2 | 22.4 | 41.1 |
| 5404 | 727 | 4.149 | 5.1 | 20.1 | 30.7 |
| 5406 | 2890 | 8.284 | 4.9 | 16.7 | 32.1 |
| 5426 | 2661 | 4.129 | 8.9 | 23.6 | 34.8 |
| 5428 | 350 | 6.464 | 2.6 | 13.1 | 44.1 |
| 5464 | 1050 | 6.21 | 2.9 | 16 | 32.4 |
| 5599 | 847 | 7.684 | 5.1 | 20.5 | 36.3 |
| 5614 | 1921 | 5.09 | 4.6 | 23.5 | 33.8 |
| 5639 | 931 | 4.265 | 4 | 13.1 | 32.5 |
| 5664 | 1271 | 3.696 | 8.5 | 16.8 | 33.1 |
| 5822 | 2728 | 7.396 | 3 | 14.4 | 33.9 |
| 5840 | 1250 | 7.793 | 14.3 | 22.7 | 31.3 |
| 5857 | 630 | 6.515 | 2.4 | 18.9 | 37.1 |
| 5899 | 1197 | 8.116 | 9.4 | 23.6 | 33 |
| 5910 | 849 | 6.287 | 9.8 | 20.5 | 41.7 |
| 5913 | 513 | 5.389 | 4.1 | 21.2 | 32.1 |
| 5922 | 2805 | 6.776 | 5.6 | 18.1 | 32.6 |
| 5926 | 545 | 3.437 | 10.1 | 23.5 | 30.9 |
| 5944 | 913 | 1.855 | 1.1 | 13.8 | 33.8 |
| 5974 | 678 | 3.478 | 7.1 | 20.2 | 30.5 |
| 6026 | 1483 | 5.025 | 7.1 | 16.9 | 32.8 |
| 6041 | 1794 | 4.182 | 11.1 | 24 | 33.6 |
| 6098 | 936 | 4.868 | 7.5 | 18.8 | 32.2 |
| 6295 | 920 | 4.558 | 12.1 | 21.3 | 36.2 |
| 6298 | 616 | 4.522 | 2.9 | 8.6 | 29.9 |
| 6303 | 592 | 3.47 | 7.9 | 24.2 | 30.7 |
| 6366 | 549 | 2.668 | 3.3 | 18.2 | 37.6 |
| 6383 | 808 | 8.97 | 7.8 | 18.8 | 31.7 |
| 6401 | 453 | 3.78 | 3.3 | 14.3 | 34.4 |
| 6406 | 470 | 5.811 | 7.9 | 16.2 | 34.6 |
| 6420 | 1128 | 5.633 | 4 | 13.2 | 32.2 |
| 6450 | 1563 | 6.934 | 6.3 | 18.2 | 32.3 |
| 6460 | 456 | 2.677 | 5.3 | 13.2 | 33.1 |
| 6462 | 1311 | 5.818 | 7 | 24.5 | 31.1 |
| 6494 | 1028 | 3.654 | 8.3 | 18 | 34.4 |
| 6500 | 1459 | 2.659 | 4.9 | 22.3 | 30.7 |
| 6639 | 467 | 2.953 | 7.5 | 18 | 27.1 |
| 6641 | 422 | 4.448 | 9 | 17.8 | 34.2 |
| 6689 | 1294 | 5.422 | 3.5 | 15.8 | 31.9 |
| 6947 | 1568 | 2.473 | 7 | 11.4 | 33.3 |
| 6992 | 557 | 2.528 | 4.1 | 17.8 | 31.1 |
| 7141 | 489 | 4.192 | 6.7 | 23.1 | 32.6 |
| 7174 | 1258 | 9.802 | 2.9 | 14.5 | 33 |
| 7313 | 982 | 5.075 | 6.7 | 22.8 | 33.3 |
| 7324 | 1111 | 3.863 | 8.2 | 16.3 | 33.4 |
| 7333 | 439 | 4.504 | 5.7 | 19.6 | 52.8 |
| 7572 | 430 | 5.15 | 3.7 | 16.5 | 35.4 |
| 7602 | 689 | 4.399 | 8.9 | 15.5 | 35.5 |
