# Supplementary material for: Resolving species boundaries in a recent radiation with the Angiosperms353 probe set: the Lomatium packardiae/L. anomalum clade of the L. triternatum (Apiaceae) complex
Source: Am J Bot. 2021 Jun 8;108(7):1217–33. doi: 10.1002/ajb2.1676 (PMC8362113; doi:10.1002/ajb2.1676)
Supplement: Supplementary file 12 — APPENDIX S12. GenBank accessions for 54 introns used in this study. [file AJB2-108-1217-s008.docx]

## Ottenlips et al.—American Journal of Botany 2021—Appendix S12

Appendix S12. GenBank accession numbers for 54 introns used in this study. Accession numbers are in the order of species, accession, GenBank numbers for introns: 4848, 4932, 4992, 5116, 5271, 5326, 5333, 5404, 5406, 5426, 5428, 5464, 5599, 5614, 5639, 5664, 5822, 5840, 5857, 5899, 5910, 5913, 5922, 5926, 5944, 5974, 6026, 6041, 6098, 6295, 6298, 6303, 6366, 6383, 6401, 6406, 6420, 6450, 6460, 6462, 6494, 6500, 6639, 6641, 6689, 6947, 6992, 7141, 7174, 7313, 7324, 7333, 7572, 7602.

***Lomatium andrusianum*; *Mansfield 16031*;** MW515874, MW515730, MW515538, MW515010, MW517170, MW516354, MW515394, MW517026, MW516402, MW515826, MW514770, MW516018, MW517122, MW515202, MW515586, MW516642, MW516834, MW514866, MW514818, MW516738, MW515682, MW517218, MW515250, MW516066, MW516114, MW515442, MW515298, MW516258, MW516306, MW516210, MW516162, MW516450, MW515778, MW516498, MW516978, MW515634, MW516882, MW517074, MW515970, MW515058, MW515922, MW517266, MW515490, MW515154, MW514914, MW516594, MW516786, MW514720, MW514962, MW516930, MW516546, MW515346, MW516690, MW515106.

***Lomatium andrusianum*; *Mansfield 16033*;** MW515858, MW515714, MW515522, MW514994, MW517154, MW516338, MW515378, MW517010, MW516386, MW515810, MW514754, MW516002, MW517106, MW515186, MW515570, MW516626, MW516818, MW514850, MW514802, MW516722, MW515666, MW517202, MW515234, MW516050, MW516098, MW515426, MW515282, MW516242, MW516290, MW516194, MW516146, MW516434, MW515762, MW516482, MW516962, MW515618, MW516866, MW517058, MW515954, MW515042, MW515906, MW517250, MW515474, MW515138, MW514898, MW516578, MW516770, MW514704, MW514946, MW516914, MW516530, MW515330, MW516674, MW515090.

***Lomatium andrusianum*; *Ottenlips 60*;** MW515866, MW515722, MW515530, MW515002, MW517162, MW516346, MW515386, MW517018, MW516394, MW515818, MW514762, MW516010, MW517114, MW515194, MW515578, MW516634, MW516826, MW514858, MW514810, MW516730, MW515674, MW517210, MW515242, MW516058, MW516106, MW515434, MW515290, MW516250, MW516298, MW516202, MW516154, MW516442, MW515770, MW516490, MW516970, MW515626, MW516874, MW517066, MW515962, MW515050, MW515914, MW517258, MW515482, MW515146, MW514906, MW516586, MW516778, MW514712, MW514954, MW516922, MW516538, MW515338, MW516682,

MW515098.

***Lomatium anomalum*; *Mansfield 16036*;** MW515869, MW515725, MW515533, MW515005, MW517165, MW516349, MW515389, MW517021, MW516397, MW515821, MW514765, MW516013, MW517117, MW515197, MW515581, MW516637, MW516829, MW514861, MW514813, MW516733, MW515677, MW517213, MW515245, MW516061, MW516109, MW515437, MW515293, MW516253, MW516301, MW516205, MW516157, MW516445, MW515773, MW516493, MW516973, MW515629, MW516877, MW517069, MW515965,

MW515053, MW515917, MW517261, MW515485, MW515149, MW514909, MW516589,

MW516781, MW514715, MW514957, MW516925, MW516541, MW515341, MW516685,

MW515101.

***Lomatium anomalum*; *Mansfield 16037*;** MW515846, MW515702, MW515510, MW514982,

MW517142, MW516326, MW515366, MW516998, MW516374, MW515798, MW514742,

MW515990, MW517094, MW515174, MW515558, MW516614, MW516806, MW514838,

MW514790, MW516710, MW515654, MW517190, MW515222, MW516038, MW516086, MW515414, MW515270, MW516230, MW516278, MW516182, MW516134, MW516422, MW515750, MW516470, MW516950, MW515606, MW516854, MW517046, MW515942,

MW515030, MW515894, MW517238, MW515462, MW515126, MW514886, MW516566,

MW516758, MW514692, MW514934, MW516902, MW516518, MW515318, MW516662,

MW515078.

***Lomatium anomalum*; *Mansfield 17017*;** MW515859, MW515715, MW515523, MW514995,

MW517155, MW516339, MW515379, MW517011, MW516387, MW515811, MW514755, MW516003, MW517107, MW515187, MW515571, MW516627, MW516819, MW514851, MW514803, MW516723, MW515667, MW517203, MW515235, MW516051, MW516099, MW515427, MW515283, MW516243, MW516291, MW516195, MW516147, MW516435,

MW515763, MW516483, MW516963, MW515619, MW516867, MW517059, MW515955,

MW515043, MW515907, MW517251, MW515475, MW515139, MW514899, MW516579,

MW516771, MW514705, MW514947, MW516915, MW516531, MW515331, MW516675,

MW515091.

***Lomatium anomalum*; *Mansfield 07-055*;** MW515852, MW515708, MW515516,MW514988, MW517148, MW516332, MW515372, MW517004, MW516380, MW515804, MW514748, MW515996, MW517100, MW515180, MW515564, MW516620, MW516812, MW514844, MW514796, MW516716, MW515660, MW517196, MW515228, MW516044, MW516092, MW515420, MW515276, MW516236, MW516284, MW516188, MW516140, MW516428, MW515756, MW516476, MW516956, MW515612, MW516860, MW517052, MW515948, MW515036, MW515900, MW517244, MW515468, MW515132, MW514892, MW516572, MW516764, MW514698, MW514940, MW516908, MW516524, MW515324, MW516668, MW515084.

***Lomatium anomalum*; *Ottenlips 45*;** MW515856, MW515712, MW515520, MW514992,

MW517152, MW516336, MW515376, MW517008, MW516384, MW515808, MW514752, MW516000, MW517104, MW515184, MW515568, MW516624, MW516816, MW514848,

MW514800, MW516720, MW515664, MW517200, MW515232, MW516048, MW516096,

MW515424, MW515280, MW516240, MW516288, MW516192, MW516144, MW516432,

MW515760, MW516480, MW516960, MW515616, MW516864, MW517056, MW515952,

MW515040, MW515904, MW517248, MW515472, MW515136, MW514896, MW516576,

MW516768, MW514702, MW514944, MW516912, MW516528, MW515328, MW516672,

MW515088.

***Lomatium anomalum*; *Ottenlips 57*;** MW515855, MW515711, MW515519, MW514991,

MW517151, MW516335, MW515375, MW517007, MW516383, MW515807, MW514751,

MW515999, MW517103, MW515183, MW515567, MW516623, MW516815, MW514847,

MW514799, MW516719, MW515663, MW517199, MW515231, MW516047, MW516095,

MW515423, MW515279, MW516239, MW516287, MW516191, MW516143, MW516431,

MW515759, MW516479, MW516959, MW515615, MW516863, MW517055, MW515951,

MW515039, MW515903, MW517247, MW515471, MW515135, MW514895, MW516575,

MW516767, MW514701, MW514943, MW516911, MW516527, MW515327, MW516671,

MW515087.

***Lomatium anomalum*; *Stevens 121*;** MW515878, MW515734, MW515542, MW515014,

MW517174, MW516358, MW515398, MW517030, MW516406, MW515830, MW514774,

MW516022, MW517126, MW515206, MW515590, MW516646, MW516838, MW514870,

MW514822, MW516742, MW515686, MW517222, MW515254, MW516070, MW516118,

MW515446, MW515302, MW516262, MW516310, MW516214, MW516166, MW516454,

MW515782, MW516502, MW516982, MW515638, MW516886, MW517078, MW515974,

MW515062, MW515926, MW517270, MW515494, MW515158, MW514918, MW516598,

MW516790, MW514724, MW514966, MW516934, MW516550, MW515350, MW516694,

MW515110.

***Lomatium anomalum*; *Stevens 123*;** MW515885, MW515741, MW515549, MW515021,

MW517181, MW516365, MW515405, MW517037, MW516413, MW515837, MW514781,

MW516029, MW517133, MW515213, MW515597, MW516653, MW516845, MW514877,

MW514829, MW516749, MW515693, MW517229, MW515261, MW516077, MW516125,

MW515453, MW515309, MW516269, MW516317, MW516221, MW516173, MW516461,

MW515789, MW516509, MW516989, MW515645, MW516893, MW517085, MW515981,

MW515069, MW515933, MW517277, MW515501, MW515165, MW514925, MW516605,

MW516797, MW514731, MW514973, MW516941, MW516557, MW515357, MW516701,

MW515117.

***Lomatium brevifolium*; *Smith 13048*;** MW515876, MW515732, MW515540, MW515012,

MW517172, MW516356, MW515396, MW517028, MW516404, MW515828, MW514772,

MW516020, MW517124, MW515204, MW515588, MW516644, MW516836, MW514868,

MW514820, MW516740, MW515684, MW517220, MW515252, MW516068, MW516116,

MW515444, MW515300, MW516260, MW516308, MW516212, MW516164, MW516452,

MW515780, MW516500, MW516980, MW515636, MW516884, MW517076, MW515972,

MW515060, MW515924, MW517268, MW515492, MW515156, MW514916, MW516596,

MW516788, MW514722, MW514964, MW516932, MW516548, MW515348, MW516692,

MW515108.

***Lomatium packardiae*; *Carlson 097*;** MW515851, MW515707, MW515515, MW514987,

MW517147, MW516331, MW515371, MW517003, MW516379, MW515803, MW514747,

MW515995, MW517099, MW515179, MW515563, MW516619, MW516811, MW514843,

MW514795, MW516715, MW515659, MW517195, MW515227, MW516043, MW516091,

MW515419, MW515275, MW516235, MW516283, MW516187, MW516139, MW516427,

MW515755, MW516475, MW516955, MW515611, MW516859, MW517051, MW515947,

MW515035, MW515899, MW517243, MW515467, MW515131, MW514891, MW516571,

MW516763, MW514697, MW514939, MW516907, MW516523, MW515323, MW516667,

MW515083.

***Lomatium packardiae*; *George 58*;** MW515848, MW515704, MW515512, MW514984,

MW517144, MW516328, MW515368, MW517000, MW516376, MW515800, MW514744,

MW515992, MW517096, MW515176, MW515560, MW516616, MW516808, MW514840,

MW514792, MW516712, MW515656, MW517192, MW515224, MW516040, MW516088,

MW515416, MW515272, MW516232, MW516280, MW516184, MW516136, MW516424,

MW515752, MW516472, MW516952, MW515608, MW516856, MW517048, MW515944,

MW515032, MW515896, MW517240, MW515464, MW515128, MW514888, MW516568,

MW516760, MW514694, MW514936, MW516904, MW516520, MW515320, MW516664,

MW515080.

***Lomatium packardiae*; *George 91*;** MW515884, MW515740, MW515548, MW515020,

MW517180, MW516364, MW515404, MW517036, MW516412, MW515836, MW514780,

MW516028, MW517132, MW515212, MW515596, MW516652, MW516844, MW514876,

MW514828, MW516748, MW515692, MW517228, MW515260, MW516076, MW516124,

MW515452, MW515308, MW516268, MW516316, MW516220, MW516172, MW516460,

MW515788, MW516508, MW516988, MW515644, MW516892, MW517084, MW515980,

MW515068, MW515932, MW517276, MW515500, MW515164, MW514924, MW516604,

MW516796, MW514730, MW514972, MW516940, MW516556, MW515356, MW516700,

MW515116.

***Lomatium packardiae*; *Mansfield 15081*;** MW515872, MW515728, MW515536, MW515008,

MW517168, MW516352, MW515392, MW517024, MW516400, MW515824, MW514768,

MW516016, MW517120, MW515200, MW515584, MW516640, MW516832, MW514864,

MW514816, MW516736, MW515680, MW517216, MW515248, MW516064, MW516112,

MW515440, MW515296, MW516256, MW516304, MW516208, MW516160, MW516448,

MW515776, MW516496, MW516976, MW515632, MW516880, MW517072, MW515968,

MW515056, MW515920, MW517264, MW515488, MW515152, MW514912, MW516592,

MW516784, MW514718, MW514960, MW516928, MW516544, MW515344, MW516688,

MW515104.

***Lomatium packardiae*; *Mansfield 15152*;** MW515870, MW515726, MW515534, MW515006,

MW517166, MW516350, MW515390, MW517022, MW516398, MW515822, MW514766,

MW516014, MW517118, MW515198, MW515582, MW516638, MW516830, MW514862,

MW514814, MW516734, MW515678, MW517214, MW515246, MW516062, MW516110,

MW515438, MW515294, MW516254, MW516302, MW516206, MW516158, MW516446,

MW515774, MW516494, MW516974, MW515630, MW516878, MW517070, MW515966,

MW515054, MW515918, MW517262, MW515486, MW515150, MW514910, MW516590,

MW516782, MW514716, MW514958, MW516926, MW516542, MW515342, MW516686,

MW515102.

***Lomatium packardiae*; *Ottenlips 20*;** MW515865, MW515721, MW515529, MW515001,

MW517161, MW516345, MW515385, MW517017, MW516393, MW515817, MW514761,

MW516009, MW517113, MW515193, MW515577, MW516633, MW516825, MW514857,

MW514809, MW516729, MW515673, MW517209, MW515241, MW516057, MW516105,

MW515433, MW515289, MW516249, MW516297, MW516201, MW516153, MW516441,

MW515769, MW516489, MW516969, MW515625, MW516873, MW517065, MW515961,

MW515049, MW515913, MW517257, MW515481, MW515145, MW514905, MW516585,

MW516777, MW514711, MW514953, MW516921, MW516537, MW515337, MW516681,

MW515097.

***Lomatium packardiae*; *Ottenlips 22*;** MW515875, MW515731, MW515539, MW515011,

MW517171, MW516355, MW515395, MW517027, MW516403, MW515827, MW514771,

MW516019, MW517123, MW515203, MW515587, MW516643, MW516835, MW514867,

MW514819, MW516739, MW515683, MW517219, MW515251, MW516067, MW516115,

MW515443, MW515299, MW516259, MW516307, MW516211, MW516163, MW516451,

MW515779, MW516499, MW516979, MW515635, MW516883, MW517075, MW515971,

MW515059, MW515923, MW517267, MW515491, MW515155, MW514915, MW516595,

MW516787, MW514721, MW514963, MW516931, MW516547, MW515347, MW516691,

MW515107.

***Lomatium packardiae*; *Ottenlips 25*;** MW515868, MW515724, MW515532, MW515004,

MW517164, MW516348, MW515388, MW517020, MW516396, MW515820, MW514764,

MW516012, MW517116, MW515196, MW515580, MW516636, MW516828, MW514860,

MW514812, MW516732, MW515676, MW517212, MW515244, MW516060, MW516108,

MW515436, MW515292, MW516252, MW516300, MW516204, MW516156, MW516444,

MW515772, MW516492, MW516972, MW515628, MW516876, MW517068, MW515964,

MW515052, MW515916, MW517260, MW515484, MW515148, MW514908, MW516588,

MW516780, MW514714, MW514956, MW516924, MW516540, MW515340, MW516684,

MW515100.

***Lomatium packardiae*; *Ottenlips 29*;** MW515877, MW515733, MW515541, MW515013,

MW517173, MW516357, MW515397, MW517029, MW516405, MW515829, MW514773,

MW516021, MW517125, MW515205, MW515589, MW516645, MW516837, MW514869,

MW514821, MW516741, MW515685, MW517221, MW515253, MW516069, MW516117,

MW515445, MW515301, MW516261, MW516309, MW516213, MW516165, MW516453,

MW515781, MW516501, MW516981, MW515637, MW516885, MW517077, MW515973,

MW515061, MW515925, MW517269, MW515493, MW515157, MW514917, MW516597,

MW516789, MW514723, MW514965, MW516933, MW516549, MW515349, MW516693,

MW515109.

***Lomatium packardiae*; *Ottenlips 32*;** MW515853, MW515709, MW515517, MW514989,

MW517149, MW516333, MW515373, MW517005, MW516381, MW515805, MW514749,

MW515997, MW517101, MW515181, MW515565, MW516621, MW516813, MW514845,

MW514797, MW516717, MW515661, MW517197, MW515229, MW516045, MW516093,

MW515421, MW515277, MW516237, MW516285, MW516189, MW516141, MW516429,

MW515757, MW516477, MW516957, MW515613, MW516861, MW517053, MW515949,

MW515037, MW515901, MW517245, MW515469, MW515133, MW514893, MW516573,

MW516765, MW514699, MW514941, MW516909, MW516525, MW515325, MW516669,

MW515085.

***Lomatium packardiae*; *Ottenlips 33*;** MW515854, MW515710, MW515518, MW514990,

MW517150, MW516334, MW515374, MW517006, MW516382, MW515806, MW514750,

MW515998, MW517102, MW515182, MW515566, MW516622, MW516814, MW514846, MW514798, MW516718, MW515662, MW517198, MW515230, MW516046, MW516094,

MW515422, MW515278, MW516238, MW516286, MW516190, MW516142, MW516430,

MW515758, MW516478, MW516958, MW515614, MW516862, MW517054, MW515950,

MW515038, MW515902, MW517246, MW515470, MW515134, MW514894, MW516574,

MW516766, MW514700, MW514942, MW516910, MW516526, MW515326, MW516670,

MW515086.

***Lomatium packardiae*; *Ottenlips 35*;** MW515891, MW515747, MW515555, MW515027,

MW517187, MW516371, MW515411, MW517043, MW516419, MW515843, MW514787,

MW516035, MW517139, MW515219, MW515603, MW516659, MW516851, MW514883,

MW514835, MW516755, MW515699, MW517235, MW515267, MW516083, MW516131,

MW515459, MW515315, MW516275, MW516323, MW516227, MW516179, MW516467,

MW515795, MW516515, MW516995, MW515651, MW516899, MW517091, MW515987,

MW515075, MW515939, MW517283, MW515507, MW515171, MW514931, MW516611,

MW516803, MW514737, MW514979, MW516947, MW516563, MW515363, MW516707,

MW515123.

***Lomatium packardiae*; *Ottenlips 36*;** MW515861, MW515717, MW515525, MW514997,

MW517157, MW516341, MW515381, MW517013, MW516389, MW515813, MW514757,

MW516005, MW517109, MW515189, MW515573, MW516629, MW516821, MW514853,

MW514805, MW516725, MW515669, MW517205, MW515237, MW516053, MW516101,

MW515429, MW515285, MW516245, MW516293, MW516197, MW516149, MW516437,

MW515765, MW516485, MW516965, MW515621, MW516869, MW517061, MW515957,

MW515045, MW515909, MW517253, MW515477, MW515141, MW514901, MW516581,

MW516773, MW514707, MW514949, MW516917, MW516533, MW515333, MW516677,

MW515093.

***Lomatium packardiae*; *Ottenlips 40*;** MW515882, MW515738, MW515546, MW515018,

MW517178, MW516362, MW515402, MW517034, MW516410, MW515834, MW514778,

MW516026, MW517130, MW515210, MW515594, MW516650, MW516842, MW514874,

MW514826, MW516746, MW515690, MW517226, MW515258, MW516074, MW516122,

MW515450, MW515306, MW516266, MW516314, MW516218, MW516170, MW516458,

MW515786, MW516506, MW516986, MW515642, MW516890, MW517082, MW515978,

MW515066, MW515930, MW517274, MW515498, MW515162, MW514922, MW516602,

MW516794, MW514728, MW514970, MW516938, MW516554, MW515354, MW516698,

MW515114.

***Lomatium packardiae*; *Ottenlips 42*;** MW515847, MW515703, MW515511, MW514983,

MW517143, MW516327, MW515367, MW516999, MW516375, MW515799, MW514743,

MW515991, MW517095, MW515175, MW515559, MW516615, MW516807, MW514839,

MW514791, MW516711, MW515655, MW517191, MW515223, MW516039, MW516087,

MW515415, MW515271, MW516231, MW516279, MW516183, MW516135, MW516423,

MW515751, MW516471, MW516951, MW515607, MW516855, MW517047, MW515943,

MW515031, MW515895, MW517239, MW515463, MW515127, MW514887, MW516567,

MW516759, MW514693, MW514935, MW516903, MW516519, MW515319, MW516663,

MW515079.

***Lomatium packardiae*; *Polito 002*;** MW515883, MW515739, MW515547, MW515019,

MW517179, MW516363, MW515403, MW517035, MW516411, MW515835, MW514779,

MW516027, MW517131, MW515211, MW515595, MW516651, MW516843, MW514875,

MW514827, MW516747, MW515691, MW517227, MW515259, MW516075, MW516123,

MW515451, MW515307, MW516267, MW516315, MW516219, MW516171, MW516459,

MW515787, MW516507, MW516987, MW515643, MW516891, MW517083, MW515979,

MW515067, MW515931, MW517275, MW515499, MW515163, MW514923, MW516603,

MW516795, MW514729, MW514971, MW516939, MW516555, MW515355, MW516699,

MW515115.

***Lomatium packardiae*; *Truksa 38*;** MW515845, MW515701, MW515509, MW514981,

MW517141, MW516325, MW515365, MW516997, MW516373, MW515797, MW514741, MW515989, MW517093, MW515173, MW515557, MW516613, MW516805, MW514837,

MW514789, MW516709, MW515653, MW517189, MW515221, MW516037, MW516085,

MW515413, MW515269, MW516229, MW516277, MW516181, MW516133, MW516421,

MW515749, MW516469, MW516949, MW515605, MW516853, MW517045, MW515941,

MW515029, MW515893, MW517237, MW515461, MW515125, MW514885, MW516565,

MW516757, MW514739, MW514933, MW516901, MW516517, MW515317, MW516661,

MW515077.

***Lomatium thompsonii*; *Ottenlips 80*;** MW515873, MW515729, MW515537, MW515009,

MW517169, MW516353, MW515393, MW517025, MW516401, MW515825, MW514769,

MW516017, MW517121, MW515201, MW515585, MW516641, MW516833, MW514865,

MW514817, MW516737, MW515681, MW517217, MW515249, MW516065, MW516113,

MW515441, MW515297, MW516257, MW516305, MW516209, MW516161, MW516449,

MW515777, MW516497, MW516977, MW515633, MW516881, MW517073, MW515969,

MW515057, MW515921, MW517265, MW515489, MW515153, MW514913, MW516593,

MW516785, MW514719, MW514961, MW516929, MW516545, MW515345, MW516689,

MW515105.

***Lomatium triternatum*; *George 102*;** MW515881, MW515737, MW515545, MW515017,

MW517177, MW516361, MW515401, MW517033, MW516409, MW515833, MW514777,

MW516025, MW517129, MW515209, MW515593, MW516649, MW516841, MW514873,

MW514825, MW516745, MW515689, MW517225, MW515257, MW516073, MW516121,

MW515449, MW515305, MW516265, MW516313, MW516217, MW516169, MW516457,

MW515785, MW516505, MW516985, MW515641, MW516889, MW517081, MW515977,

MW515065, MW515929, MW517273, MW515497, MW515161, MW514921, MW516601,

MW516793, MW514727, MW514969, MW516937, MW516553, MW515353, MW516697,

MW515113.

***Lomatium triternatum*; *Lesica 10541*;** MW515890, MW515746, MW515554, MW515026,

MW517186, MW516370, MW515410, MW517042, MW516418, MW515842, MW514786,

MW516034, MW517138, MW515218, MW515602, MW516658, MW516850, MW514882,

MW514834, MW516754, MW515698, MW517234, MW515266, MW516082, MW516130,

MW515458, MW515314, MW516274, MW516322, MW516226, MW516178, MW516466,

MW515794, MW516514, MW516994, MW515650, MW516898, MW517090, MW515986,

MW515074, MW515938, MW517282, MW515506, MW515170, MW514930, MW516610,

MW516802, MW514736, MW514978, MW516946, MW516562, MW515362, MW516706,

MW515122.

***Lomatium triternatum*; *Lesica 10552*;** MW515880, MW515736, MW515544, MW515016,

MW517176, MW516360, MW515400, MW517032, MW516408, MW515832, MW514776,

MW516024, MW517128, MW515208, MW515592, MW516648, MW516840, MW514872,

MW514824, MW516744, MW515688, MW517224, MW515256, MW516072, MW516120,

MW515448, MW515304, MW516264, MW516312, MW516216, MW516168, MW516456,

MW515784, MW516504, MW516984, MW515640, MW516888, MW517080, MW515976,

MW515064, MW515928, MW517272, MW515496, MW515160, MW514920, MW516600,

MW516792, MW514726, MW514968, MW516936, MW516552, MW515352, MW516696,

MW515112.

***Lomatium triternatum*; *Lesica 10794*;** MW515850, MW515706, MW515514, MW514986,

MW517146, MW516330, MW515370, MW517002, MW516378, MW515802, MW514746,

MW515994, MW517098, MW515178, MW515562, MW516618, MW516810, MW514842,

MW514794, MW516714, MW515658, MW517194, MW515226, MW516042, MW516090,

MW515418, MW515274, MW516234, MW516282, MW516186, MW516138, MW516426,

MW515754, MW516474, MW516954, MW515610, MW516858, MW517050, MW515946,

MW515034, MW515898, MW517242, MW515466, MW515130, MW514890, MW516570,

MW516762, MW514696, MW514938, MW516906, MW516522, MW515322, MW516666,

MW515082.

***Lomatium triternatum*; *Lesica 10798*;** MW515844, MW515700, MW515508, MW514980,

MW517140, MW516324, MW515364, MW516996, MW516372, MW515796, MW514740,

MW515988, MW517092, MW515172, MW515556, MW516612, MW516804, MW514836,

MW514788, MW516708, MW515652, MW517188, MW515220, MW516036, MW516084,

MW515412, MW515268, MW516228, MW516276, MW516180, MW516132, MW516420,

MW515748, MW516468, MW516948, MW515604, MW516852, MW517044, MW515940,

MW515028, MW515892, MW517236, MW515460, MW515124, MW514884, MW516564,

MW516756, MW514738, MW514932, MW516900, MW516516, MW515316, MW516660,

MW515076.

***Lomatium triternatum*; *Mansfield 16064*;** MW515887, MW515743, MW515551, MW515023, MW517183, MW516367, MW515407, MW517039, MW516415, MW515839,

MW514783, MW516031, MW517135, MW515215, MW515599, MW516655, MW516847,

MW514879, MW514831, MW516751, MW515695, MW517231, MW515263, MW516079,

MW516127, MW515455, MW515311, MW516271, MW516319, MW516223, MW516175,

MW516463, MW515791, MW516511, MW516991, MW515647, MW516895, MW517087,

MW515983, MW515071, MW515935, MW517279, MW515503, MW515167, MW514927,

MW516607, MW516799, MW514733, MW514975, MW516943, MW516559, MW515359,

MW516703, MW515119.

***Lomatium triternatum*; *Mansfield 16078*;** MW515886, MW515742, MW515550, MW515022, MW517182, MW516366, MW515406, MW517038, MW516414, MW515838,

MW514782, MW516030, MW517134, MW515214, MW515598, MW516654, MW516846,

MW514878, MW514830, MW516750, MW515694, MW517230, MW515262, MW516078,

MW516126, MW515454, MW515310, MW516270, MW516318, MW516222, MW516174,

MW516462, MW515790, MW516510, MW516990, MW515646, MW516894, MW517086,

MW515982, MW515070, MW515934, MW517278, MW515502, MW515166, MW514926,

MW516606, MW516798, MW514732, MW514974, MW516942, MW516558, MW515358,

MW516702, MW515118.

***Lomatium triternatum*; *Mansfield 16082*;** MW515863, MW515719, MW515527, MW514999, MW517159, MW516343, MW515383, MW517015, MW516391, MW515815, MW514759, MW516007, MW517111, MW515191, MW515575, MW516631, MW516823, MW514855, MW514807, MW516727, MW515671, MW517207, MW515239, MW516055, MW516103, MW515431, MW515287, MW516247, MW516295, MW516199, MW516151, MW516439, MW515767, MW516487, MW516967, MW515623, MW516871, MW517063, MW515959, MW515047, MW515911, MW517255, MW515479, MW515143, MW514903, MW516583, MW516775, MW514709, MW514951, MW516919, MW516535, MW515335, MW516679, MW515095.

***Lomatium triternatum*; *Ottenlips 59*;** MW515857, MW515713, MW515521, MW514993, MW517153, MW516337, MW515377, MW517009, MW516385, MW515809, MW514753, MW516001, MW517105, MW515185, MW515569, MW516625, MW516817, MW514849, MW514801, MW516721, MW515665, MW517201, MW515233, MW516049, MW516097, MW515425, MW515281, MW516241, MW516289, MW516193, MW516145, MW516433, MW515761, MW516481, MW516961, MW515617, MW516865, MW517057, MW515953, MW515041, MW515905, MW517249, MW515473, MW515137, MW514897, MW516577, MW516769, MW514703, MW514945, MW516913, MW516529, MW515329, MW516673, MW515089.

***Lomatium triternatum*; *Ottenlips 62*;** MW515888, MW515744, MW515552, MW515024,

MW517184, MW516368, MW515408, MW517040, MW516416, MW515840, MW514784,

MW516032, MW517136, MW515216, MW515600, MW516656, MW516848, MW514880,

MW514832, MW516752, MW515696, MW517232, MW515264, MW516080, MW516128,

MW515456, MW515312, MW516272, MW516320, MW516224, MW516176, MW516464,

MW515792, MW516512, MW516992, MW515648, MW516896, MW517088, MW515984,

MW515072, MW515936, MW517280, MW515504, MW515168, MW514928, MW516608,

MW516800, MW514734, MW514976, MW516944, MW516560, MW515360, MW516704,

MW515120.

***Lomatium triternatum*; *Ottenlips 65*;** MW515862, MW515718, MW515526, MW514998,

MW517158, MW516342, MW515382, MW517014, MW516390, MW515814, MW514758,

MW516006, MW517110, MW515190, MW515574, MW516630, MW516822, MW514854,

MW514806, MW516726, MW515670, MW517206, MW515238, MW516054, MW516102,

MW515430, MW515286, MW516246, MW516294, MW516198, MW516150, MW516438,

MW515766, MW516486, MW516966, MW515622, MW516870, MW517062, MW515958,

MW515046, MW515910, MW517254, MW515478, MW515142, MW514902, MW516582,

MW516774, MW514708, MW514950, MW516918, MW516534, MW515334, MW516678,

MW515094.

***Lomatium triternatum*; *Ottenlips 69*;** MW515871, MW515727, MW515535, MW515007,

MW517167, MW516351, MW515391, MW517023, MW516399, MW515823, MW514767,

MW516015, MW517119, MW515199, MW515583, MW516639, MW516831, MW514863,

MW514815, MW516735, MW515679, MW517215, MW515247, MW516063, MW516111,

MW515439, MW515295, MW516255, MW516303, MW516207, MW516159, MW516447,

MW515775, MW516495, MW516975, MW515631, MW516879, MW517071, MW515967,

MW515055, MW515919, MW517263, MW515487, MW515151, MW514911, MW516591,

MW516783, MW514717, MW514959, MW516927, MW516543, MW515343, MW516687,

MW515103.

***Lomatium triternatum*; *Ottenlips 72*;** MW515860, MW515716, MW515524, MW514996,

MW517156, MW516340, MW515380, MW517012, MW516388, MW515812, MW514756,

MW516004, MW517108, MW515188, MW515572, MW516628, MW516820, MW514852,

MW514804, MW516724, MW515668, MW517204, MW515236, MW516052, MW516100,

MW515428, MW515284, MW516244, MW516292, MW516196, MW516148, MW516436,

MW515764, MW516484, MW516964, MW515620, MW516868, MW517060, MW515956,

MW515044, MW515908, MW517252, MW515476, MW515140, MW514900, MW516580,

MW516772, MW514706, MW514948, MW516916, MW516532, MW515332, MW516676,

MW515092.

***Lomatium triternatum*; *Ottenlips 73*;** MW515879, MW515735, MW515543, MW515015,

MW517175, MW516359, MW515399, MW517031, MW516407, MW515831, MW514775,

MW516023, MW517127, MW515207, MW515591, MW516647, MW516839, MW514871,

MW514823, MW516743, MW515687, MW517223, MW515255, MW516071, MW516119,

MW515447, MW515303, MW516263, MW516311, MW516215, MW516167, MW516455,

MW515783, MW516503, MW516983, MW515639, MW516887, MW517079, MW515975,

MW515063, MW515927, MW517271, MW515495, MW515159, MW514919, MW516599,

MW516791, MW514725, MW514967, MW516935, MW516551, MW515351, MW516695,

MW515111.

***Lomatium triternatum*; *Ottenlips 74*;** MW515889, MW515745, MW515553, MW515025,

MW517185, MW516369, MW515409, MW517041, MW516417, MW515841, MW514785,

MW516033, MW517137, MW515217, MW515601, MW516657, MW516849, MW514881,

MW514833, MW516753, MW515697, MW517233, MW515265, MW516081, MW516129,

MW515457, MW515313, MW516273, MW516321, MW516225, MW516177, MW516465,

MW515793, MW516513, MW516993, MW515649, MW516897, MW517089, MW515985,

MW515073, MW515937, MW517281, MW515505, MW515169, MW514929, MW516609,

MW516801, MW514735, MW514977, MW516945, MW516561, MW515361, MW516705,

MW515121.

***Lomatium triternatum*; *Ottenlips 76*;** MW515867, MW515723, MW515531, MW515003,

MW517163, MW516347, MW515387, MW517019, MW516395, MW515819, MW514763,

MW516011, MW517115, MW515195, MW515579, MW516635, MW516827, MW514859,

MW514811, MW516731, MW515675, MW517211, MW515243, MW516059, MW516107,

MW515435, MW515291, MW516251, MW516299, MW516203, MW516155, MW516443,

MW515771, MW516491, MW516971, MW515627, MW516875, MW517067, MW515963,

MW515051, MW515915, MW517259, MW515483, MW515147, MW514907, MW516587,

MW516779, MW514713, MW514955, MW516923, MW516539, MW515339, MW516683,

MW515099.

***Lomatium triternatum*; *Ottenlips 77*;** MW515864, MW515720, MW515528, MW515000,

MW517160, MW516344, MW515384, MW517016, MW516392, MW515816, MW514760,

MW516008, MW517112, MW515192, MW515576, MW516632, MW516824, MW514856,

MW514808, MW516728, MW515672, MW517208, MW515240, MW516056, MW516104,

MW515432, MW515288, MW516248, MW516296, MW516200, MW516152, MW516440,

MW515768, MW516488, MW516968, MW515624, MW516872, MW517064, MW515960,

MW515048, MW515912, MW517256, MW515480, MW515144, MW514904, MW516584,

MW516776, MW514710, MW514952, MW516920, MW516536, MW515336, MW516680,

MW515096.

***Lomatium triternatum*; *Smith 10748*;** MW515849, MW515705, MW515513, MW514985,

MW517145, MW516329, MW515369, MW517001, MW516377, MW515801, MW514745,

MW515993, MW517097, MW515177, MW515561, MW516617, MW516809, MW514841,

MW514793, MW516713, MW515657, MW517193, MW515225, MW516041, MW516089,

MW515417, MW515273, MW516233, MW516281, MW516185, MW516137, MW516425,

MW515753, MW516473, MW516953, MW515609, MW516857, MW517049, MW515945,

MW515033, MW515897, MW517241, MW515465, MW515129, MW514889, MW516569,

MW516761, MW514695, MW514937, MW516905, MW516521, MW515321, MW516665,

MW515081.
